# The power of the group – Group-based parenting programmes for disadvantaged parents and their infants: a realist review

**DOI:** 10.1016/j.ijnsa.2026.100591

**Published:** 2026-06-10

**Authors:** Rachel Verweij, Jo Howe, Jolanda Mathijssen, Hedwig van Bakel

**Affiliations:** a Tilburg University, Tranzo Center for care and wellbeing, Prof. Cobbenhagenlaan 125, 5037DB Tilburg, the Netherlands; b Aston University, Birmingham, School of pharmacy, B4 7RT Birmingham, UK; c The Realist Hub Ltd, 11 Charlotte Way, Atherstone, Warwickshire VC9 1AS, UK; d Open Universiteit, Department of Clinical Psychology, Valkenburgerweg 177, 6419AT Heerlen, the Netherlands

**Keywords:** Disadvantaged parents, Parenting education, Parent-child relations, Infant, Social support, Social participation, Parenting programmes

## Abstract

**Purpose:**

Despite growing evidence of the importance of supporting disadvantaged parents during the first phase of parenthood, most existing parenting programmes targeted at this population are individually delivered, even though group-based programmes are cost-efficient, require fewer personnel (i.e. specialised nurses), and are appreciated by parents, particularly those experiencing social isolation. Given the potential benefits, the aim of this study is to understand how and under what circumstances group-based programmes can work effectively for disadvantaged populations.

**Methods:**

We conducted a five-stage realist review to identify the contexts and mechanisms underpinning the delivery of parenting programmes for disadvantaged parents of infants. An initial programme theory was developed. A formal literature search was conducted across five bibliographic databases, supplemented by a Google search and expert advice to identify programmes. Three expert advisory groups comprising parents and professionals played a key role in shaping the review’s direction and refining the programme theories.

**Results:**

We identified five group-based parenting programmes specifically designed for disadvantaged parents of infants. Twenty-eight documents (scientific and grey literature) were analysed, leading to the development of sixteen context-mechanism-outcome configurations, which were synthesised within six programme theories. These theories were presented within an overarching diagram highlighting the contextual factors and mechanisms which influence parental learning and development. The results were that a non-judgmental, supportive approach and the creation of a safe group space helped parents relax and engage. Parents reported being able to reflect and reframe past experiences, learn from peers and facilitators, strengthen multiple relationships, feel less isolated and feel closer to their infant. Reported outcomes extended beyond improved parent-child interaction and included the development of new support networks, engagement with education and employment, and social participation.

**Conclusion:**

Group-based parenting programmes can support disadvantaged parents of infants when they are delivered in ways that align with parents’ lived circumstances and social histories. Supportive and non-judgmental group environments appear to trigger mechanisms of trust, emotional safety, and the normalising of shared experiences, enabling parents to engage, reflect on past experiences and learn from peers and facilitators. These mechanisms are more likely to operate when practical and relational barriers to participation (such as mistrust of services, transport difficulties) are addressed. Outcomes extended beyond parenting confidence and parent-infant interaction to include reduced isolation and wider social participation. Future research should focus on how these mechanisms are sustained over time and how they shape longer-term outcomes for parents and children across differing service contexts.

**What is already known?**

• Disadvantaged parents often face multiple, intersecting challenges that limit engagement with parenting programmes, including social isolation, poor mental health, and distrust of services.
• Parenting support for disadvantaged parents of infants is more commonly delivered through individually based, nurse-led home visiting programmes, which are resource intensive and difficult to scale.

Alt-text: Unlabelled box dummy alt text

**What this adds**

• This realist review synthesises evidence from five group-based parenting programmes for disadvantaged parents of infants to develop an overarching programme theory underpinned by 16 context–mechanism–outcome configurations.
• The findings explain how safe, non-judgmental group environments and skilled facilitation help reduce isolation, build trust, and support parental reflection, learning, and confidence.
• The review shows that group processes are central mechanisms of change, with impacts extending beyond parenting practices to include improved wellbeing, empowerment, social participation, and engagement with wider services.

Alt-text: Unlabelled box dummy alt text

## 1 Introduction

Sensitive, supportive and stimulating parenting is central to promoting a child’s development, fostering physical, social, emotional and psychological well-being into adult life (Britto et al., 2017). Pregnancy and the first two years of life in particular, are a sensitive period during which a great part of the brain's structure and capacity is shaped (Cusick & Georgieff, 2016; Nelson et al., 2019). Poverty, particularly when experienced early and over a sustained period, is a key stressor linked to suboptimal perinatal and early development, later social and behavioural difficulties and educational outcomes (Brown et al., 2012; Chaudry & Wimer, 2016; Vos et al., 2014).

Poverty is a widespread risk factor for suboptimal development: in the Netherlands, 6,8% of households live in poverty and almost one in ten children grow up in such a household (Centraal Plan Bureau, 2021, 2022). In other western countries these numbers range from under 5% of children in Finland and Denmark, to over 20% in Spain, Israel and the USA, averaging 12,8% in all OECD countries, with the UK and Australia close to average (13.6 and 13.3%) (OECD data explorer, 2025)*.*

Poverty is often interlinked with other risk factors, such as unemployment, social isolation, low parental education, housing problems, and young or single parenthood, supporting the use of the broader term ‘socioeconomic deprivation’ or ‘disadvantage’ (Hamza et al., 2024). The link between disadvantage and child outcomes is thought to stem from the way disadvantage limits the practical and emotional support parents can offer, making it harder for them to focus on and respond appropriately to their infant’s needs (Lovejoy et al., 2000; Parkes et al., 2016; Stein et al., 2014), and increasing chances of child maltreatment and neglect (Skinner et al., 2023). Also, research has established a causal relationship between poverty and mental illness. Worries and uncertainty about volatile income and expenditures, or living in inadequate housing in low-income neighborhoods, can worsen mental health (Ridley et al., 2020).

Nevertheless, many children living in disadvantaged circumstances do well (Kiernan & Mensah, 2011). Sensitive and nurturing relationships within the early years, and good parental mental health, have been shown to mitigate the long-term impact of disadvantage (Kiernan & Mensah, 2011; Parkes et al., 2016; Smith et al., 2023). There is increasing consensus that public investment in universal access to high-quality services, particularly within areas of higher socioeconomic deprivation, is required in order to reach the most vulnerable children and promote health equity (Black et al., 2017; Marmot et al., 2012). A targeted approach seems needed—one that not only supports parenting skills and the parent-child relationship, but also addresses underlying social and emotional challenges such as financial stress and isolation (Butler et al., 2020; Rosenblum et al., 2018; Whittaker & Cowley, 2012). Well-researched individualised nurse-led home visiting programmes, such as ‘Nurse Family Partnership’ and ‘Minding the Baby’, provide this type of combined support during pregnancy and in the first year with a baby. These programmes benefit disadvantaged families (van Assen et al., 2020; Kitzman et al., 2019; Molloy et al., 2021; Rayce et al., 2017), but the usefulness of individualised programs may be compromised by heavy financial and personnel demands (Cunningham et al., 1995; Gilmer et al., 2016; Torres et al., 2011).

Group-based parenting programmes may be a less resource intensive alternative to home visiting: they are generally considered cost efficient and effective (Michelson et al., 2013; Wittkowski et al., 2016), and are associated with improvements in parental psychosocial wellbeing, parental self-efficacy (Wittkowski et al., 2016), as well as the behavioural and emotional adjustment of children (Barlow et al., 2014, 2016). When these programmes are delivered within community settings, this may offer additional benefits to families, reducing social isolation and facilitating community building (Butler et al., 2020). However, most group parenting programs with a substantial evidence base, such as ‘Incredible Years’ and the ‘Triple P program’, tend to focus on parental management of toddler or school children’s behavior, not on improving positive parenting of babies (Barlow et al., 2014, 2016; Jones et al., 2016). Also, meta-analyses suggest that disadvantaged parents are less likely to enroll in these programmes and their children show poorer programme outcomes (Lundahl et al., 2006; Reyno & McGrath, 2006).

### 1.1 Present study

Evaluating and understanding how and why preventative early parenting group-based programmes work, is crucial for decision makers and service providers aiming to develop, embed and sustain effective support for disadvantaged parents (Gilmer et al., 2016; Kilburn et al., 2017; Leviton & Trujillo, 2017). One such programme is the Cuddle & Care programme developed in the Netherlands, designed specifically for disadvantaged parents with (or expecting) an infant (*Cuddle & Care | Impluz*, n.d.). Although early feedback from parents and programme providers in the pilot phase of the program has been positive (among others, asking 10 participating mothers how they experienced the group using a brief questionnaire), little is known about the underlying processes through which programmes like Cuddle & Care achieve their outcomes.

The study aimed to identify group-based parenting programmes for disadvantaged parents of infants and then to generate explanations as to how and why these programmes worked (or did not work) to influence parental well-being and the parent-infant relationship. The findings are intended to inform both broader service development and the ongoing refinement of the Cuddle & Care programme, which will be evaluated in a separate research (currently ongoing).

## 2 Methods

Realist review is well suited to evaluating complex programmes, because it goes beyond asking whether a programme works, to explore how, why, for whom, and under what circumstances it works. This approach is particularly valuable when programmes are implemented in varied contexts and produce different outcomes across populations (Wong et al., 2013). By unpacking the underlying mechanisms and contextual factors, a realist review can generate nuanced, actionable insights to inform policy and practice. Realist reviews produce testable (either confirming, refining or refuting) programme theories: the central explanatory ideas that describe how and why an intervention works (or doesn’t), for whom, and under what circumstances. These are underpinned by context-mechanism-outcome configurations (hereafter referred to as ‘configurations’), which offer more granular detail. These configurations specify how, in a given context, programme resources trigger participant reasoning (mechanism) to produce a behavioural outcome or result (foreseen or unforeseen) (Pawson et al., 2005a).

Between 2022 and 2025, we conducted a five-stage realist review (Pawson et al., 2005b). Due to the volume of data, and in line with RAMESES guidance (see supplementary file 1) and established practice in realist reviews, the scope was refined through a process of progressive focusing, guided by relevance to the emerging programme theory rather than exhaustive coverage of all possible interventions or studies (Booth et al., 2020; Howe, 2024; Howe et al., 2023; Wong et al., 2013).

For the overall Cuddle & Care project three stakeholder groups guided the research process. Institutional ethics approval (TSB_RP433) was obtained, including the current review and the evaluation of the Cuddle & Care mother-baby groups (to be published separately).

Group 1, the expert group, consisted of three academics (with expertise on poverty, social work and infant mental health), two patient and public representatives, and two of the programme developers (professionals working in the field of Infant Mental Health), recruited from personal networks. Meetings with Group 1 occurred approximately every six months for the entire duration of the research.
Group 2, the lived experience group, comprised mothers with lived experience, recruited from the six participating Cuddle and Care groups. Three online meetings were convened with Group 2. Attendees could fluctuate; in total respectively five, four and six mothers attended.
Group 3 consisted of group facilitators from the seven participating Cuddle & Care groups, plus one from an organisation with the intention of starting a group. In total, this group met six times, mostly live.

### 2.1 Stage 1: focus, initial programme theory

An initial programme theory (supplementary file 2) was developed. In line with RAMESES guidance, Stage 1 involved an exploratory scoping literature search, the aim of which was to build familiarity with the topic area, identify relevant parenting programmes, and inform development of the initial programme theory, rather than a formal scoping literature review (Booth et al., 2020), and by three interviews with mothers who participated in former Cuddle & Care groups, the project team knowledge, and input from the Group 1 stakeholder group.

### 2.2 Stage 2: literature search

The literature search was split into three phases:

#### 2.2.1 Phase 1, identifying programmes

Phase 1 identified group parenting programmes for disadvantaged parents with children under two years of age and any related literature. At this point of the study, the unit of inclusion was the parenting programme rather than individual evaluation studies.

A formal literature search was conducted in PsycINFO, PubMed, Web of Science, Cochrane, Social Services Abstract and Proquest. An academic librarian aided in the formation of search strings (supplementary file 3). The initial programme theory, alongside input from the expert stakeholder group, helped to determine the key concepts utilised within the search strategy. The search was subsequently broadened, as no literature was found when disadvantage was factored in as a search term. Searching was iterative: additional articles were found by snowballing (reviewing reference lists of key papers), citation chaining (forward citation tracking), Google searching (using search words similar to the ones used in the formal search) and consultation with subject matter experts (lecturers specialising in poverty and social work, professionals working with disadvantaged parents, client representatives).

In line with realist guidance (Wong et al., 2013), Phase 1 involved iterative and purposeful consultations with a small number of academics and professionals (approximately four) to refine programme identification and theory development. This consultation informed focus decisions rather than generating primary data. These individuals were identified through professional networks and consulted on an ad hoc basis to help identify relevant programmes and assess their relevance to disadvantaged parents of infants. This consultation group was distinct from the formal expert stakeholder group convened to guide the review overall, although some individuals contributed in both capacities. Consistent with RAMESES guidance (Wong et al., 2013), the purpose of this consultation was to inform theory development and focus decisions rather than to generate primary data, and therefore precise participant numbers were not fixed in advance.

From the literature, programmes were identified, and information was gathered on their content.

#### 2.2.2 Phase 2, narrowing the scope with expert stakeholders

Phase 2 involved selecting the most relevant programmes. In a meeting with the expert stakeholder group, criteria were formulated and prioritised to help the research team to select the programmes that most closely fit the goals and characteristics of the Cuddle & Care programme. This was done using an online tool. The most important selection criteria were:

→ Started before the toddler age (2 years)
→ Did not stop after the baby was born (prenatal)
→ Targeting the broad group of disadvantaged parents (low socioeconomic status, but no specific (clinical) subgroup)

There were no strict exclusion criteria, rather the best fitting programmes were selected. Results of the selection process were discussed and agreed upon in the next meeting.

#### 2.2.3 Phase 3, expanding the search / identifying more papers from the selected programmes

Additional searches were conducted to identify all published material (peer reviewed and grey) relating to each selected programme. Each database used in phase 1 was searched again, using the exact name of the parenting programme (e.g., Mellow Babies). Additionally, a Google search was conducted to identify websites relating to the programmes and any additional articles that were not identified via the database searches. Corresponding authors from key academic papers and relevant charitable organisations were contacted to assist in the identification of additional published material, including grey literature, for example, poster presentations and web pages. Documents were organised in a referencing database with corresponding identification tags indicating source (for example, database, corresponding authors, charitable organisation etc.).

### 2.3 Stage 3: selection and appraisal of documents

All titles and abstracts were screened by the first author (RV) for relevance to the programme theory, with a 10% sample independently screened by an associate researcher. Articles were excluded if they did not contain any relevant details about one of the selected parenting programmes, could not be retrieved, parents were not physically present (i.e. incarcerated), only contained a study protocol, or were written in a language other than English.

All articles were assessed at full text for relevance and richness (the rating process is described in detail in supplementary file 4), with a ten percent sample independently assessed in duplicate by RV, JM, and HVB. Discrepancies were resolved via discussions. Documents with a rating of relevant and ‘rich’, and relevant and ‘medium rich’, were included in the review (Dada et al., 2023). Articles were rated based on the depth and explanatory value of the data they contained in relation to contexts, mechanisms, and outcomes relevant to parenting programmes for disadvantaged parents. The initial programme theory was used as a sensitising framework to guide early appraisal; however, the list of mechanisms was illustrative rather than exhaustive, and documents were also assessed for their capacity to inform mechanisms not anticipated in the initial theory (Wong, 2013).

### 2.4 Stage 4: data extraction, analysis

Characteristics of included articles were extracted into Microsoft Word and incorporated into a table. Included documents were imported into Atlas Ti for data extraction and analysis. Data extraction focused on information with explanatory relevance to the review aim. Specifically, data were extracted on (i) intervention characteristics and delivery features (e.g., programme structure, facilitation practices); (ii) participant responses, reasoning and experiences, interpreted as potential mechanisms; and (iv) reported outcomes at individual, relational and social levels.

Relevant extracts were coded against a coding framework (see supplementary file 5) developed by RV and discussed with JM and HVB. The coding framework was initially informed by the initial programme theory and included deductive codes relating to engagement, trust, facilitator role, group processes, parental reflection, and parent-infant interaction. The framework was tested for suitability using two included articles and subsequently revised. Inductive coding was undertaken alongside framework-based coding to capture new information not anticipated in the initial framework. In realist analysis these inductive codes often reflect the emerging insights into individuals’ reasoning or sense-making (mechanism), descriptions of conditions or circumstances (context), or explanations of why programmes did or did not work in particular circumstances. Coding was undertaken to support the development and refinement of the programme theory. Extracted data was compared within and across documents to test, refine and synthesise the context-mechanism-outcome configurations. Coding was undertaken by RV, with regular discussion within the research team to refine interpretations and support transparency of analytic judgements. This combined inductive–deductive approach is consistent with RAMESES guidance for realist reviews, which emphasises iterative, theory-driven analysis rather than the application of a fixed qualitative coding protocol (Wong et al., 2013).

### 2.5 Stage 5: data synthesis, configuration development and programme theory refinement

Coded data was exported into Microsoft Word. Tentative configurations were developed, summarized in programme theories, and shared regularly with the research team, alongside extracted quotes. They were also validated with the lived experience group and a focus group of group facilitators, in an iterative process.

## 3 Results

**Fig. 1:**
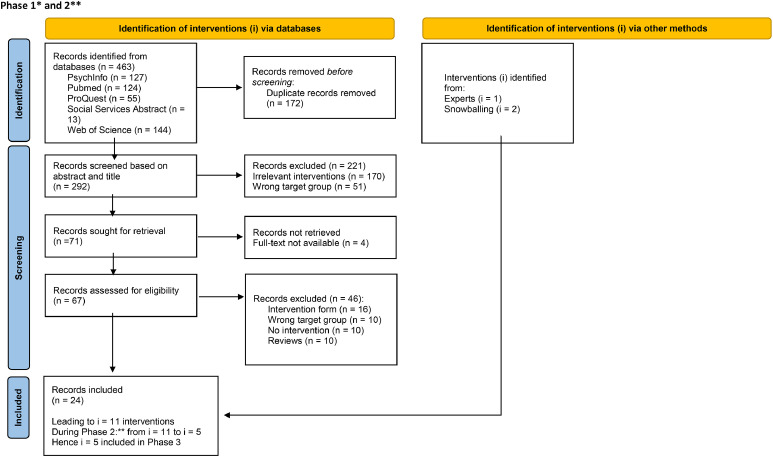

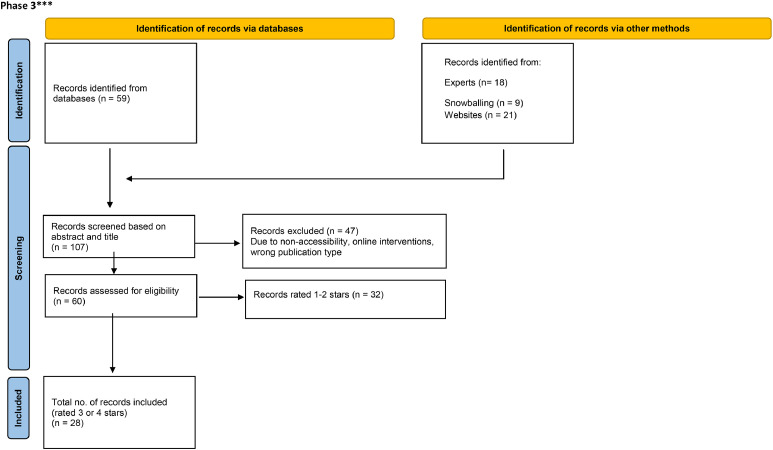
Flow Diagram of the literature search for a realist review of group-based parenting programmes.

Stage 1. The initial program theory that informed the analysis, is provided in supplementary file 2.
Stage 2. The results of the literature search are presented in Fig. 1. In phase 1292 papers were identified. Following screening, eight parenting programmes were identified (N = 24 documents). An additional three programmes were identified via experts and snowballing (evidence sources included websites and grey material). In total eleven programmes were identified.

Detailed analysis of the eleven parenting programmes was undertaken by the researchers and the expert advisory group to identify programmes most relevant to the programme theory. In phase 2 the scope was narrowed, and six parenting programmes were excluded as they did not specifically target disadvantaged parents or included mothers with older children. The five included programmes are: Mellow Babies (UK), Parent Infant Program (Ireland), Young parents’ programme and Mother Baby Nurture Programme (Both from Australia) and Baby and Us (UK). Characteristics of these five programmes are summarised in Table 1.

**Table 1:** complete list of parenting programmes, selected and non-selected.

Name/ founded	Targeted parents	Goal of program	Results	Strategies to facilitate enrollment		Programme Content							
				Lowering threshold	Other	Frequency/ duration	Group leader	Help with finances	Parent-baby interaction	Physical contact	Mental health	Partner role	Group size
C&C* 2019 NL	At-risk parents. children <30 mo.	Reduce stress; Improve parental wellbeing and parent-infant attachment	As yet unknown	Free and ‘soft’ entry, parallel childcare or baby in group, food, delivery in community centers. Home visits, support calls/ app	Referral; Help with social services; liaison officer.	40-120 weekly 2 h sessions	1-2 social workers, half-day trained	Yes, provided by local organisation	IMH-specialist; Group discussion, co-designed	Yes, baby-wearing	Differs per group, co-designed	Partner or family meetings sometimes	6-10 mothers or mother-baby dyads
MB* 1994 UK	Parents with psychosocial difficulties and babies <18 mo.	Improve parent-baby interaction, parental mental health and parenting confidence; Prevent conduct disorder and reduce child protection concern	Parental satisfaction with programme; Lower depression and anxiety scores; Improved parenting skills and parenting confidence; Improved positive interactions / attachment/ perceived connectedness; De-escalation child protection services; Improved child social/ cognitive development; Reduced barrier to health services	Free entry, taxi transport, parallel childcare and food; Low literacy materials	Referral	14 weekly 5 h sessions,	2 per group, 3-day trained/ supervised professionals with experience with young parents, male leader for dad groups	No	Video feedback; Parent-baby play; Coaching; Homework; Infant focused speech	Sometimes baby massage	Yes: intensive psycho education and counseling on dealing with anxiety/ depression	Separate fathers’ or mothers’ groups.	6-8
PIN* 2014 EI	Mothers from disadvantaged areas (35-55% low-income), babies 2-24 mo.	Improve parent competency and wellbeing, parent-infant relationships and child outcomes	Parental satisfaction with programme; Increased parenting confidence/ efficacy and wellbeing; Stress reduction; Improvements in parent-baby interaction, positive parenting, child development and pro-social behaviours; Parents reported feeling supported and better understanding infant needs	Home visits, support calls, introductory meetings; Baby in group, so no childcare needed.	Help with other social services; Referral	15-20 weekly 2-3 hour sessions, then break, 10 toddler sessions	2 PHN + 1 community worker, trained/coached in wraparound care	No	Psycho education; Parent-baby activities/ play	Yes, baby massage	Psycho education on selfcare and support	No, only mothers	8-10
YPP* 2014 AU	Mothers <25 years, disadvantaged area, babies < 36 mo.	Improve parenting skills and help to attend school	Improved parenting skills, knowledge and confidence; Network and link to community services	Free and ‘soft’ entry, delivery in community centers, baby in group, support calls/ app, home visits	Referral	50-120 weekly 2 h sessions	Team of different disciplines	Yes	Parent/ baby play; Group discussion	Yes, baby massage	Not known	No, only mothers	8-20
MBN* 2020 AU	Distressed (disadvantaged) mothers, 310 yearly, babies<6 m	Improve parent-infant relationship; Improve mentalization capacity	Improved mentalizing; Decreased depression, anxiety and parenting stress	Free entry, baby in group, aboriginal liaison officer	Referral	10 weekly 2 h sessions	2 early parenting professionals, 3-day trained and supervise	No, parents must be financially stable before entry.	Parent-baby play; Group discussion; Infant focused speech	Promoting touch during meetings	No	No, only mothers	6 mother-baby dyads
B&U* 2020 UK	Socially disadvantaged parents with infants <12 mo.	Improve parent-infant relationship, positive parenting, parent self-esteem and parenting confidence	Parental satisfaction with programme; Self-efficacy; Mental wellbeing; Confidence and parenting skills; Goal attainment; Decreased behaviour problems	Free and open entry, peer led in community centers, baby in group	Introductory meeting	8 weekly 2,5 h sessions	Two local parents, 7,5-day trained and supervised	No	Psycho education; Group discussion	No	Psycho education; Group discussion	Mixed, but 98% mothers	6-14 parent-baby dyads
CP 1995 USA	All parents of babies 1-24 mo., including disadvantaged	Improve health care, parental empowerment and network; Promote (racial) health equity	High attendance; Improved clinical outcomes (e.g.: vaccination); Parental and clinician satisfaction with programme; Parental self-efficacy; Cost reduction (compared to well-baby care as usual)	Free entry	Midwifery care delivered in-group	15 2 h sessions over the first 4 years	Physician or nurse	No	Psycho education; Group discussion	No	Psycho education; Group discussion	One parent can join	5-10 parent-baby dyads
MomP202010	Mothers with trauma, mental health problems, children <5yo	Mitigate impact of stress/ trauma; Improve wellbeing; Enhance sensitive parenting	High completion rates: 65-70%; Reduced depression and PTSD scores; Improved parenting confidence, social support and connection to care; Improved maternal representations	Free entry, transport, childcare and food.	Not known	10 weekly 3 h meetings + 3 home visits	Two community clinicians, 1 university level, both 3-day trained	Linking to care if needed	Psycho education; Guided parent-baby interactions	No	Psycho education on selfcare and support	Parenting partner invited to 1 meeting	Not known
CHUGS	Depressed mothers with infants <1 y	Improve parent-baby relationship	Parental satisfaction with programme; Improvements in parent-baby interaction	Not known	Not known	10 2 h meetings	Not known	Not known	Play, music	Not known	Psycho education	Not known	Not known
COSP 2007 USA	All parents	Improve attachment	Improved attachment classification and quality of caregiving; Improved caregiver self-efficacy; Decreased caregiver depression scores; No or small effects in at-risk parents (parenting distress).	No	No	8 sessions	QCF-6 therapist (university), 4-day trained	No	Reflective videos of parenting, Explaining visual attachment model (COS)	No	Not known	Differs	Not known
FF 2015 Finland / 2002 USA	Universal, integrated in health care system	Not known	Peer support; parents report better understanding of baby and more insights into self as parent; Improved parent-child interaction; Family involvement	Free entry	No	2-weekly sessions, in total 12 2 h sessions	2 social or health care workers, 4-day trained and supervised	No	Reflective questioning /contemplation; Observing baby	No	Not known	Whole family (mother father baby)	Not known
RFTS UK 1985	Not known	Improved parent-baby attachment	Small improvements in attachment and sensitivity (equal to Home Start)	Free entry, transport,	Not known	8 2 h sessions	Infant development specialists	Not known	Discussing parent-child videos	Not known	Not known	Not known	12-40, split in small groups

Abbreviations:

PHN = Public Health Nurse

IMH = Infant Mental Health

‘soft entry’ = entry at any time point, continuous group, no referral or indication needed

C&C: Cuddle & Care Programme

MB = Mellow Babies (based on MP: Mellow parenting)

B&U = Empowering Parents, Empowering Communities, version: Baby and Us

PIN = Infant and Parent Program (based on the Incredible Years Program, but contains extras and is longer)

YPP = Young Parents Program

MBN = Mother Baby Nurture Program

CP = Centering Parenting

FF = Families First (based on Parents First)

COSP = Circle of Security Parenting

RftS = Right from the Start

MomP = Mom Power

In phase 3, 107 records were identified for the five selected parenting programmes, including the 24 found earlier.

Stage 3. All records were screened and 60 articles remained, 28 of which were deemed relevant for developing configurations. Reported outcomes included increased parenting confidence, improved parental mental health, more positive parenting practices, strengthened parent-infant relationships, reduced involvement with child protection services and better connections to community support. Most outcomes were based on self-reports, qualitative studies and/or or pre-post intervention designs, with three small non-randomised control trials identified (Hickey, 2015; Puckering et al., 2010).

**Fig. 2:**
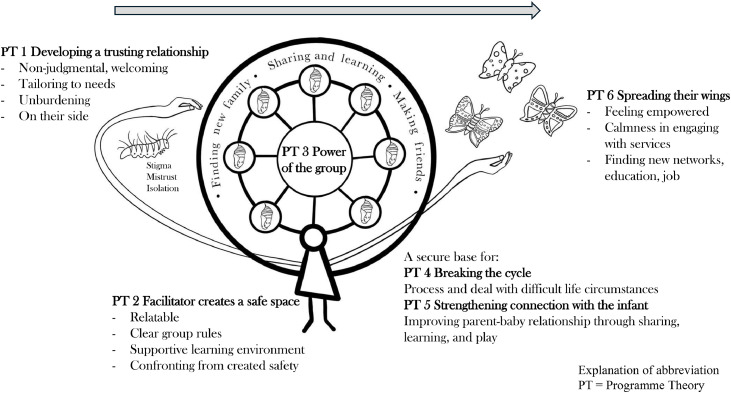
Overarching diagram of the six Programme Theories.

In stage 4 and 5, all 28 papers were read (characteristics of the papers are to be found in supplementary file 6) and analysed and six programme theories underpinned by 16 configurations were constructed (see supplementary file 7). In some cases, a rival theory was formulated, indicated by an ‘R’ in the numbering. An overarching diagram of the six programme theories is provided in Fig. 2.

Programme theories 1 and 2 relate to the early stage of the parent’s journey, focusing on how they are introduced to the facilitator and begin to engage with the ethos and purpose of the group. Programme theory 3 focuses on the group experience itself, which sits at the heart of the programmes. Programme theories 4 and 5 also unfold during the group process, exploring how particular forms of support, connection, and reflection can shape parents’ experiences and outcomes. Programme theory 6 considers what happens as the group ends, including how parents make sense of their journey, and how changes may be carried forward into everyday life.

The imagery of the caterpillar, cocoon, and butterfly is used to symbolise the change and, in some cases the transformation, that many parents and facilitators describe over the course of the programmes that they have been part of.

A description, interspersed with quotations, of each of the six programme theories can be found below. The programme theories synthesise multiple configurations identified across the dataset; configurations are not presented as standalone theories but as empirical configurations that underpin and refine each programme theory. A list of all 16 configurations can be found in supplementary file 7. Quotations are followed by the title of the specific programme that they came from.

### 3.1 Programme theory 1: developing a trusting relationship

In whatever way disadvantage presents, poverty, low literacy, mental health problems, teenage parenthood, minority status and/or violence or addiction, many parents report feeling ashamed, stigmatised, judged and undervalued, both in their own social environment and by the professionals responsible for their care. These experiences, often compounded by histories of insecure attachment, contribute to distrust of authorities and services designed to support them, and the fear that their child may be removed from their care, which can influence participation in parenting groups (configuration 1).

When parenting groups are introduced in contexts of vulnerability and mistrust among parents, enrolment is more likely to occur if group leaders invest time and effort to foster trust through relational, non-judgmental, and welcoming practices, including the use of trusted intermediaries such as peer parents, midwives, public health nurses, or community workers (configuration 2). Trust is generated when parents perceive that group leaders are “on their side”, understand their lived realities, and offer practical and emotional support rather than assessment or judgement. Trust is further strengthened when programmes are adapted to the specific need of parents, such as the need for social connection, mental health support and self-care (configuration 3), and provide practical solutions for barriers to attending, particularly in contexts of limited financial or material resources (configuration 4).

When these contextual and relational conditions are present, parents are more able to relax, lower their defences and be open to the resources the parenting group has to offer, increasing enrolment and enabling openness to participation and learning (configuration 5).

Illustrative evidence:

Feelings of stigma and fear of judgement were commonly reported as barriers to engagement:

*Feelings of stigmatisation or guilt appear to disproportionately affect at-risk parents… further discouraging them from engaging in parenting interventions due to their fear of being judged* (Davidson et al., 2023). [Mellow Babies]

Investments were needed to help mitigate these barriers:

*The programme addresses … barriers to attendance (such as fear, stigma or childcare/transport issues) and aims to build trustworthy relationships with service providers* (Hickey et al., 2024). [Parent Infant Programme]

### 3.2 Programme theory 2: facilitator creates a ‘safe space’

Programme theory 2 proposes that parents’ engagement and learning are enabled when group facilitators, such as health care nurses, psychologists, community workers, and/or trained peer parents (see Table 1), have sufficient interpersonal skills to create a true connection and a psychologically safe and inclusive group environment. In contexts where parents have experienced judgement, exclusion, or surveillance, feeling free to speak without fear of criticism is a prerequisite for participation and reflection. When parents feel welcomed, valued, and accepted as they are, trust and self-confidence increase. This supports respectful group interactions, adherence to group norms, improved parenting practices, and more constructive communication with services, including those previously feared, such as child protection (configuration 6). A sense of safety is also fostered when facilitators are perceived as relatable, credible, and respectful. Facilitators who share characteristics or experiences with parents—such as parenthood, community background, ethnicity, or gender are more readily trusted. This can help parents engage in the group process, including accepting challenge and reflection when needed (configuration 7). Facilitators themselves report learning through these relationships, developing deeper respect for parents’ lived experiences (configuration 8).

Illustrative evidence:

Facilitators’ consistent and accepting stance was described as providing a corrective relational experience:

*By maintaining a consistent, thoughtful, compassionate, and accepting stance, facilitators offer a potentially new experience for mothers where these qualities may have been longed for but not experienced ... with their own caregivers* (Cooke et al., 2023). [Mother Baby Nurture]

The credibility and inclusiveness of peer and community-based facilitators further supported engagement:

*“Parents knowing that [group leaders] are willing to stand up and be the spokesperson and support families, it reaches out to those people and includes them*” (NES Early Intervention Framework). [Baby and Us]

### 3.3 Programme theory 3: finding a new family – the power of the group

Programme theory 3 explains how the group itself becomes a central mechanism of change. Once parents, after initial fear and hesitancy, come to understand that the group is a safe space and that other parents share similar experiences, feelings of social and emotional isolation begin to diminish. Through shared stories and mutual recognition, parents begin to value their own and others’ experiences, trust peer feedback, and recognise their own parenting strengths.

Many parents come to experience the group as something qualitatively different from a formal programme setting — it takes on the character of a new family. Beyond friendly relations, parents describe bonds of unconditional acceptance, connectedness, and mutual support. These family-like qualities — feeling known, valued, and supported — offer a sense of belonging that many parents had previously lacked, and may lead to broader social participation for parents and their children (configuration 9)

However, for parents with severe social anxiety or mental health challenges, group participation may remain difficult, and differences within groups or lack of external support can limit engagement (configuration 9R).

This peer validation supports confidence, openness to learning, and willingness to reflect on parenting practices. Over time, the authenticity and reciprocity of group interactions foster a deeper understanding of social relationships and belonging (configuration 10).

Illustrative evidence:

Parents frequently described the group as a source of belonging and emotional relief:

*Coming here is like time out — it is like stress relief…I just can’t wait to get here to meet with my classmates because they are like sisters now* (Penehira & Doherty, 2012). [Mellow Babies]

Peer learning was actively encouraged and valued:

*You’d have one mum share what worked, then another, and suddenly you have a group of people trying to help you* (Strange et al., 2019). [Young Parent Program]

### 3.4 Programme theory 4: breaking the cycle

Programme theory 4 proposes that when groups provide a secure and supportive environment, they form a safe base for parents to reflect on past (often traumatic) experiences and reinterpret their parenting histories (configuration 11). If programmes focus solely on skills, without acknowledging parents’ personal histories and current life stressors, then parenting stress may continue to hinder the emotional attunement that programmes aim to support (configuration 11R).

When space is made for reflection, parents report feeling understood and validated. This acknowledgement helps parents become more open to change, recognise maladaptive behaviors, improve multiple relationships in their lives (including those within the group), and experience increased confidence in parenting and improvements in mental health and self-understanding. This reflective process may contribute to interrupting intergenerational cycles of trauma by enabling parents to parent differently than they were parented (configuration 12).

Illustrative evidence:

The group functioned as a containing and reflective environment:

*The group process acts as a holding environment .... When a member of the group shares an affective state, the containing experience … can be amplified and nuanced as the multiple members provide a “hall of mirrors” response* (Cooke et al., 2023). [Mother Baby Nurture]

Parents described the relief of sharing and reframing difficult experiences:

*You’re realising it’s not just you that’s been through bad stuff and sharing that with somebody, how you felt - is really comforting* (Davidson et al., 2023). [Mellow Babies]

### 3.5 Programme theory 5: strengthening connection with the infant

Programme theory 5 explains how group participation supports both infant development and the parent–infant relationship. In a stimulating and supportive group environment, the social world of both parent and infant begins to expand. Parents observe infant development, and receive positive reinforcement from facilitators, peers, and infants themselves, reducing parenting stress and increasing confidence (configuration 13). They also exchange advice and learn practical skills such as playing or reading to the infant and reading cues in an informal way. This helps parents take a more proactive role in their infants development and supports greater emotional attunement (configuration 14). Notably, these changes seem to occur in groups where infants are present as active participants as well as where parallel childcare is provided. Over time, parents report deeper feelings of connection, attachment, and enjoyment in their relationship with their infant. (configuration 15)

The first year with an infant seems a particularly good window of change, for reasons of neuroplasticity and newness of the parenting role.

Illustrative evidence:

Facilitators described how observing infant development was a powerful encouragement for parent’s change:

*Huge, really huge. Because the children’s behaviour had improved, their level of hassle, their level of stress around parenting reduced quite significantly (*Penehira & Doherty, 2012). [Mellow Babies]

Facilitators’ role-modelling and positive affirmation of parent–infant interaction supported bonding:

*Just having someone there to say, “She’s looking at you. She needs you… you’re a great mum,” … promotes attachment and caring and bonding* (Strange et al., 2019). [Young Parent Program]

### 3.6 Programme theory 6: spreading their wings

Programme theory 6 proposes that the group functions as a bridge to wider society. From the secure base of the newly found relationships within the group, parents often develop confidence to maintain relationships, seek new support networks, and engage with services that were previously avoided or feared. The trust they developed can generalise to other contexts, enabling parents to access healthcare and community resources and sometimes even education or employment, reframing future possibilities for themselves and their children (configuration 16).

However, for others, the standard duration of group-based programmes may be insufficient to sustain these changes. The end of the group can reintroduce feelings of loss or insecurity, indicating the need for longer-term or stepped support and effective referral pathways (configuration 16R).

Illustrative evidence:

Familiarity with services reduced fear and increased access:

*That demystifies .. this health centre… and that fear factor is taken from them* (Hickey et al., 2021). [Parent Infant Program]

Parents described renewed aspiration and motivation:

*This group said, “You can get a job and make your daughter proud”… no-one’s ever said that. No-one’s ever pushed me towards doing it* (Davidson et al., 2023). [Mellow Babies]

## 4 Discussion

In this paper, we performed a realist review of group-based parenting programmes for disadvantaged parents and their infants. Five programmes from three countries were found. We looked for the underlying mechanisms of these programmes: why, for whom, and in which circumstances, can these programmes work to achieve the desired effects of improvements in parental wellbeing, parent-infant interaction, and ultimately child development?

The results revealed six general programme theories, that we summarise and discuss below.

**Programme theory 1** identified the importance of actively addressing distrust towards service providers, often rooted in past experiences, fear of child removal and/or histories of unsafe relationships. Previous studies have shown that marginalised groups, such as ethnic minorities, often face discrimination and inequities in both access to, and quality of, healthcare, making distrust a logical, and even adaptive, response when they find themselves in vulnerable situations (Griffith et al., 2021). As demonstrated by our findings, understanding and addressing this distrust is necessary to regain people’s trust. Previous research has also identified high rates of insecure attachment styles amongst at-risk individuals, who may display higher levels of anxiety and be more fearful of new experiences (P. Thomson & Jaque, 2017). The context of the parents in the five included programmes suggests the possibility of a history of both negative experiences with services, and of unsafe relationships. These factors may also interact and reinforce each other.

Parenting programmes often struggle to engage disadvantaged parents (Gonzalez et al., 2018), and do not use a specific engagement strategy (Gilmer et al., 2016). The five programmes identified here show that it *is* possible to reach and involve parents, even in highly challenging circumstances. Key to this success were community-based approaches, using trusted intermediaries, addressing distrust towards services, and adopting a welcoming, low-threshold, non-judgmental, and non-stigmatising attitude. By lowering barriers to participation, these programmes created more accessible and supportive environments.

In scientific literature, common suggested strategies for recruiting underserved populations align with our findings, including community-based and participatory approaches, word of mouth recruitment, use of multiple recruitment channels, and facilitating attendance (e.g. transportation) (Mendoza-Vasconez et al., 2016).

**Programme theory 2** emphasised the crucial role of the group facilitator, not only in supporting parents’ initial engagement (as highlighted in programme theory 1), but also in creating a supportive group space where parents feel safe to open and learn. All five programmes share key components: a focus on parents’ strengths, hands-on practical learning and delivery in a group-based format. The personality and interpersonal skills of the group facilitator are crucial to creating and fostering this safe, inclusive learning environment. These findings align with a small number of previous process evaluations of parent-training programmes, which have shown that parents’ feelings of support within the group and their perceived alliance with the facilitator, are important factors in effectiveness (Akin et al., 2018).

Although the five programmes varied in several respects including frequency and duration (see Table 1), education of facilitators (ranging from peers to psychotherapists), use of video feedback and the degree of attention given to parents’ personal history, this did not appear to influence the overall outcomes. Across the programmes, parents consistently reported increases in parenting confidence and more positive parent-child interactions. Furthermore, parents tended to value the interpersonal qualities of the facilitator more than the specific programme components.

Interestingly, in the field of psychotherapy, decades of research indicate that common factors across therapies, such as empathy, warmth, and the collaborative therapeutic relationship or ‘alliance’ correlate more highly with client outcome than the exact treatment intervention applied (Lambert & Barley, 2001). The quality of the therapeutic alliance is widely recognised as a robust predictor of outcomes across different psychotherapies (Wampold & Flückiger, 2023). Although much of this theory and research originates from the domain of psychotherapy, the concept is applicable to any practice involving a person ‘seeking help from a socially sanctioned healer’ (Wampold & Flückiger, 2023).

Task-sharing and peer-led approaches may further enhance accessibility, particularly for low income and socially excluded families, as seen in the Baby and Us Programme. (Harwood et al., 2022).

**Programme theory 3** described the group process as central to the success of these programmes. Participating in a group not only helps fill in gaps in the social network of disadvantaged parents but also empowers and provides learning opportunities. These arise by normalising and mirroring shared experiences, and by instilling hope through mutual support.

These findings resonate with previous research on social support programmes for diverse groups ranging from diabetes to tinnitus to postpartum women, describing ‘social support’, ‘sense of belonging’, ‘being understood’, ‘non-judgment’, ‘receiving reassurance’, and ‘gaining a stronger sense of self-worth’ as helpful aspects (Borek et al., 2019a; 2019b; Dennis, 2010; Pryce et al., 2019; Small et al., 2011)*.* Similarly, disadvantaged women engaged in group-based prenatal and postnatal care have also described group environments as a safe haven, characterised by respectful, non-judgmental spaces that allowed them to share and support each other whilst learning new skills (Hackley et al., 2019). Compared to traditional one-on-one prenatal care, group care has been associated with improved outcomes, such as patient satisfaction with providers (Friedman et al., 2021), increased engagement with other services, reductions in preterm birth, higher condom use (Ickovics et al., 2016), improved breastfeeding and smoking cessation and psychological outcomes (Byerley & Haas, 2017). These observations echo core mechanisms of change identified in group therapy, where participants often describe relief from recognising the universality of their experience, feeling hopeful after hearing others’ stories and benefitting from corrective relational and emotive experiences from the facilitators (Lothstein & Thomas, 2021).

Some papers included in this review reference the use of online platforms (e.g. WhatsApp or Facebook) as a low-threshold way to create support. These digital spaces allowed mothers to ask questions and receive support at any time of the day or night. However, there was insufficient evidence to develop a full configuration on the role of social media in supporting group processes and easing facilitator burden. Nevertheless, this represents an interesting avenue for future research.

**Programme theory 4** focused on creating opportunities within the group to talk about past trauma and current life difficulties, helping parents develop a new perspective on their life stories, become aware of past mistakes and maladaptive behaviors both of themselves, their parents, and their (past) romantic partners, and find new ways of coping with present stressors. This aligns with existing evidence suggesting that the provision of reflective opportunities may help prevent the intergenerational transmission of disadvantage (Schoon & Melis, 2019).

Based on our findings, we would argue that parents in disadvantaged circumstances may require more time to build trust in the facilitator and the group. They may need space to process past traumas or current problems before they are ready to focus on improving their mental health or relationships with others, including their infants.

This may have important implications for the ongoing debate about the optimal type, format and intensity of universal early parenting programmes. Shorter (6–8 meetings) and more focused programmes have often been recommended as more effective for changing parental behaviour (Bakermans-Kranenburg et al., 2005, 2023; Leijten et al., 2022; Lindsay & Totsika, 2017), but these appear to have limited impact on parental mental health or children’s emotional difficulties (Leijten et al., 2018). Some authors have argued for moving away from named or ‘branded’ programmes towards more adaptive approaches, that better meet the individual needs of parents (Hickey et al., 2023).

**Programme theory 5** found that concentrating on the infant’s development and capacities offers a positive and non-threatening way for parents to talk about bonding with their child and parenting skills. Experiences with psychodynamic toddler groups have also shown that mothers who are supported and provided with child developmental information about their toddlers’ developing minds, are better able to resolve typical infancy and toddlerhood difficulties (*Mother-Baby-Toddler Group Guide*, n.d.).

It seems reasonable to expect that infants would benefit from the increase in wellbeing, personal growth and empowerment reported by parents, alongside the exchange of parenting advice and hands-on practice in the group.

However, the evidence base for these programmes remains relatively limited, and future research is required to confirm the nature and extent of their impact on parent-infant interaction and infant development. Only three small non-randomised control trials were identified, one of which evaluates the Mellow Babies Programme, reporting a clinically significant decrease in maternal depression scores as well as significant improvements in mother-infant interaction, whilst the control group showed increased depression scores and decreased interaction scores (Hickey, 2015; Puckering et al., 2010). In general, little is known about the efficacy of parent education interventions (Gilmer et al., 2016).

**Programme theory 6** found that, for many parents, their time in the group creates a secure base making it possible to regain trust and find their way to other services, social networks, education and/or a job. The concept of epistemic trust (trust in communicated knowledge) (Li et al., 2023) may help understand this process: once parents start to believe that information from other participants is valid, trustworthy and relevant, this belief may transfer toward other sources of information. It has been suggested that epistemic trust asks for a letting down of our default epistemic vigilance (Fonagy & Allison, 2023; Knapen et al., 2020), which may be seen as the opposite of the mistrust towards authorities and services that many parents bring to the group.

It is worth noting that these programmes promote much broader outcomes than child development. Their goals include empowering parents, strengthening communities, and addressing health inequities, potentially helping to break the cycle of intergenerational deprivation.

Similarly, participation in group antenatal health care for the general population was found to enable access to a community, especially for women new to the area, and stimulate the spreading of health knowledge to family members and into the wider community (Horn et al., 2025).

It would be interesting to follow parents over a longer period to explore the progress they make after the group ends. As far as we could find, no long-term follow-up or longitudinal research on any of the programmes exists.

### 4.1 Stakeholder reflections

All stakeholder groups reflected on the conclusions during their meetings. There was a lot of recognition of common themes, for example the context of participating parents and the investment needed to help them attend (programme theory 1), the importance of the group in the lives of parents and labelling the group as ‘family’ (programme theory 3), mothers expressing their delight and confidence when facilitators or other mothers labelled their babies’ development in a positive way (programme theory 5) and how many parents find their way to jobs or study (programme theory 6). Some specific remarks made were:

The expert group highlighted that creating a safe space (programme theory 2) can be quite a responsibility for facilitators, especially peer facilitators - or those with heavy time restraints, i.e. health care nurses. Safeguarding concerns were also highlighted and these are echoed in the literature, where peer facilitators have expressed uncertainty about their safe guarding competence (Thomson et al., 2015). Suggestions made included sharing responsibility by encouraging mothers to support each other and trusting this process and organising regular supervision and peer-to-peer reflection.

The facilitator and lived experience group noted that discussing trauma (programme theory 4) in the context of new parents entering the group regularly, may require a separate time and space to discuss difficult topics in a more contained group, and only when parents feel ready for that. They also suggested spreading this conversation over several meetings, to allow parents to re-think and come back to the content.

## 5 Strengths and limitations

A strength of this review is the inclusion of an expert stakeholder group (including patient and public representatives), a specific parent group and a group consisting of group facilitators. These groups were consulted at key points during the review process and helped to develop and validate the programme theory and context-mechnism-outcome configurations.

A key limitation of this study relates to the number of published programmes targeting disadvantaged parents. Although more programmes may exist, we were not able to retrieve their results. Although we were able to extract six programme theories from the literature and they were deemed relevant by the expert group, professionals and parents, nevertheless we based these theories on just 28 papers and five programmes. The lack of long-term follow-up for these programmes means that we cannot determine the long-term outcomes of these groups. Next, we did not find much information on parents that chose not to join a programme, and their reasons for that. Lastly, it can be expected that different backgrounds or training of group facilitators may impact results, not in the least because of possible differences in their ability to engage with trauma. However, we did not find enough data to robustly develop a configuration on this subject.

## 6 Conclusion and recommendations

We identified 16 configurations and merged them into 6 programme theories, explaining how safe group processes create a sense of belonging and lift isolation, helping parents open up, learn and make connections into wider society. Key parameters for success include the quality of the group facilitator to help counteract previous negative experiences, a non-judgmental and supportive approach, facilitation of a safe space for the group learning process, and openness in the program to discuss parent’s questions, needs and difficulties.

These configurations and programme theories are currently being evaluated within an accompanying realist evaluation of the Dutch Cuddle & Care project, which will be published in due course.

Low-threshold, group-based parenting programmes are a promising avenue for supporting parents and babies living in disadvantaged circumstances. Knowledge and clarity about underlying mechanisms and (potentially) active ingredients of early parenting intervention may help policy makers understand when group-based parenting programmes may be a good choice for underserved communities, and guide decisions about developing programmes and tailoring them to parents’ needs.

## CRediT authorship contribution statement

**Rachel Verweij:** Writing – review & editing, Writing – original draft, Visualization, Investigation, Funding acquisition, Formal analysis, Conceptualization. **Jo Howe:** Writing – review & editing, Methodology, Formal analysis, Data curation. **Jolanda Mathijssen:** Writing – review & editing, Validation, Supervision, Data curation. **Hedwig van Bakel:** Writing – review & editing, Supervision, Project administration, Funding acquisition, Conceptualization.

## Declaration of competing interest

The authors declare the following financial interests/personal relationships which may be considered as potential competing interests:

Rachel Verweij reports financial support was provided by Netherlands Organisation for Health Research and Development. Jo Howe received consulting fees from Tilburg University for her contribution to this article. If there are other authors, they declare that they have no known competing financial interests or personal relationships that could have appeared to influence the work reported in this paper.

## Appendix Supplementary materials

Image, application 1

Image, application 2

Image, application 3

Image, application 4

Image, application 5

Image, application 6

Image, application 7
