# Supplementary material for: The power of the group – Group-based parenting programmes for disadvantaged parents and their infants: a realist review
Source: Int J Nurs Stud Adv. 2026 Jun 10;11:100591. doi: 10.1016/j.ijnsa.2026.100591 (PMC13320447; doi:10.1016/j.ijnsa.2026.100591)
Supplement: Supplementary file 2 [file mmc2.docx]

**Supplementary File 2: Initial Programme Theories**


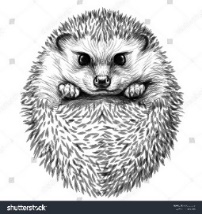


Group supervisor/ community organisation actively picks them up (puts a lot of work into that!), possibly together with peer (and/or referrers i.e. midwives, doctor, youth health care). Supervisor is someone with ALL of these:

- Expertise on group process (social worker, youth worker or similar, no health worker)
- Expertise on target group (poverty, ethnicity, language, reaching and engaging)
- Personality ('tigress', fights for 'her' mothers, long-term commitment, provides powerful leadership, charisma, committed, puts in a lot of time)
- She builds bridges (with the medical/ mental health field, referrers, financial and other content experts) (PS this works both ways, they help each other, learn and grow)
- There is continuity (she is supported, including financially, by her organization or municipality)

(*PS: Questions: who are those she does NOT reach? How universal is this intervention, if so dependent on such a person.*

And helps them toward:

1 Stability

2 The group

Maslov Pyramid

First housing, relational or financial problems,

then ‘higher’ goals i.e. parenting, work or study

**
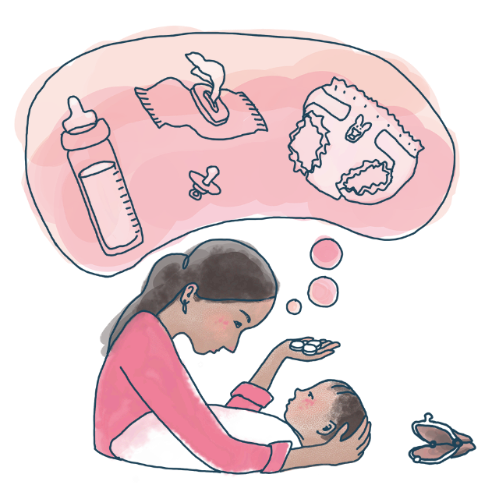
INITIAL PROGRAM THEORIES**

The initial theories (hypotheses) we describe here are based on:

- the original underpinning of the intervention Cuddle & Care

- a small initial exploratory literature review

- three interviews with participants

and will be confirmed, falsified and/or refined throughout the research process.

Short explanation of Realist Research: Context + Mechanism = Outcome, and Mechanism consists of 2 things: Mechanism resource and Mechanism response.

**Theories related to the various components of the project**

*Financial support*

1) When parents are poor (have little financial resources and/or debt), this leads to a lot of paperwork, stress and little time and attention for their baby (context). Practical help and information in the group by the financial expert and a positively experienced group discussion that breaks taboos around financial problems (mechanism resource) lead to stress reduction through increased understanding and the realisation that they may apply for a grant or tax returns (mechanism response).

*parent-baby support*

*
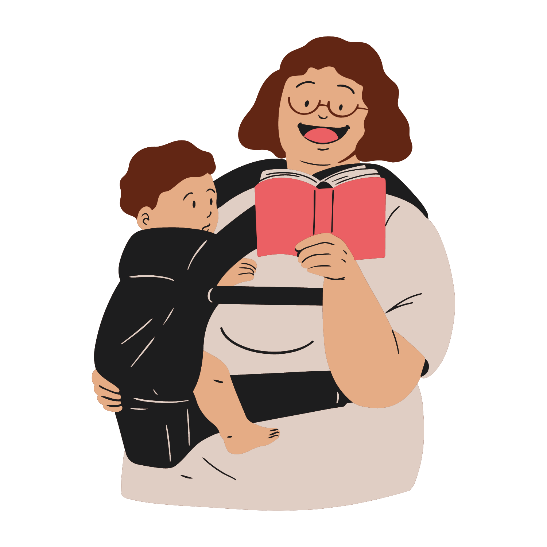
*2) If mothers themselves have an insecure attachment history and are not used to responsive parenting themselves (context), information by the IMH expert, and a positively experienced group discussion about daily problems, for example around crying, sleeping and eating, (mechanism resource) can help mothers to see the baby's needs separately from their own history, and learn to trust what the baby indicates (mechanism response). This leads to a better parent-child relationship (outcome).

*Convenient babywearing*

3) If mothers have little time, for example because they are single and/or still studying or combining work and household (context), babywearing will make it easier for them to combine tasks with taking care of their baby's needs (mechanism resource); this will increase the closeness between mother and child (mechanism response) and thus bonding/attachment and indirectly parental well-being (outcome).

*Intimate babywearing*

4) When parents with a positive cognition about babywearing (context) carry their baby (mechanism resource), this creates hormonal changes, such as an increase in "maternal hormones" prolactin and oxytocin and a decrease in stress hormones (mechanism response), and this leads to more positive feelings about babies and to more attention to the babies' needs (mechanism response) and this in turn leads to an increase in well-being and parent-child relationship (outcome).

*Uncomfortable babywearing*

5) In mothers who themselves have an avoidant or chaotic attachment style (context), babywearing (mechanism resource) will increase feelings of discomfort about being so close to the baby and they will project this onto their babies, thinking that their babies don't like being so close or "confined”. This will result in mothers either not using the baby carrier or using it in a more distant and practical way (for transportation reasons) (mechanism response). As a result, the intended effect on the parent-baby relationship will not occur (outcome).

*Eating together*

6) Eating together in the Cuddle & Care group (mechanism resource) leads to a specific kind of connectedness (mechanism response), because eating is something that everyone has to do and is connected to all kinds of aspects of the culture you grew up in and also because while doing practical things together such as cooking and during eating together, conversations arise about other subjects (mechanism response), and this connectedness increases group loyalty, the formation of a social network and that in turn leads to well-being and parent-child relationship (outcome).

*Health*

7) Among parents with low income and/or debt (context), eating together in the group (mechanism resource) leads to more healthy meals and a higher sense of physical wellbeing (outcome).

**
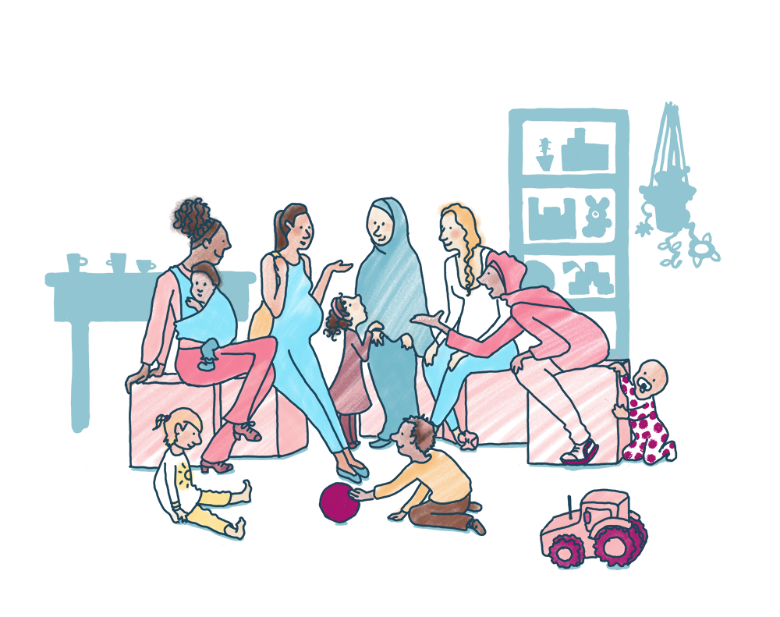
**

**Theories related to the project as a whole and to the group**

*Feeling welcome*

8) If mothers have low self-confidence and confidence in themselves as parents (context), the invitation for, being a part of, and the support of the group in Cuddle & Care (mechanism resource), will make them feel that they matter in society, their baby matters, that they are welcome and that they matter as a parent (mechanism response) and this will positively affect well-being and (through more effective parenting strategies) parent-child relationship (outcome).

*Group facilitator*

9) If mothers have few social skills (context), the presence of a group facilitator with appropriate training, a lot of experience with and a sense for group processes (mechanism resource) leads to learning new skills such as dealing with conflict and building friendships (mechanism response), which leads to parents growing in self-confidence, which in turn leads to increased well-being and a better parent-child relationship (outcome).

*Network*

10) When mothers with little social network, especially single mothers, (context) participate in a group with other mothers they can get to know and trust a group where both positive and negative experiences and emotions are shared (mechanism resource), and this gives them the opportunity to gain positive experience with friendship and build a network so they can get help and support (mechanism response), which makes them grow in self-confidence (outcome)

*Learning from others*

11) When insecure mothers with little social network participate (context), it gives them the opportunity to learn on the spot from other mothers (mechanism resource), and see that they are not alone, that they are not doing so badly, and the chance to also help others with their experience (m. response) which makes them grow in their parenting and self-confidence (outcome).

*Overcoming distrust*

12) If parents have distrust in counseling due to bad previous experiences (context), getting to know trustworthy, non-judgmental and approachable counselors/group leaders (mechanism resource) will lead to a more general increase in trust in counseling and daring to ask for help (mechanism response) and this will lead to easier referral to additional help if needed and this will lead to an increase in well-being and parent-child relationship (outcome). OR (rival theory) they will not really engage themselves and not show up.

*Multi-problems*

13) If the problems of parents are large and lie in several areas of life (context), they will attend the meetings less often, they will be less successful in building a network and they will be less able to adapt their behavior (mechanism), so Cuddle & Care will not lead to the desired effect (outcome).

**Theories related to uptake and even the starting of a group (the right conditions)**

*Bridging the culture gap*

14) If the target group of vulnerable mother/mothers in poverty is distrustful towards social care and has its own language and (sub)culture, which (higher class) social care providers do not always speak (context), mothers will not come to the group (outcome), unless there are intermediaries, who speak the language and understand the culture (for example, come from the neighborhood themselves) (mechanism resource). This will enable mothers to trust Cuddle and Care (mechanism response) and start participating (outcome).

*Bridging the organizational gap*

15) The structure of care in municipalities is often fragmented, with little contact between, on the one hand, youth care, where there is a lot of knowledge on the young child, but often focused on one-on-one counseling, and on the other hand, the primary field (social work/ neighborhood care/ community care) where people are used to working with groups, but are not so focused on the baby age (context). Innovative structures are needed, such as independent foundations, or youth professionals or municipal employees who know how to build bridges (mechanism resource), to enable new C&C groups (mechanism response/ outcome).
