# Supplementary material for: The power of the group – Group-based parenting programmes for disadvantaged parents and their infants: a realist review
Source: Int J Nurs Stud Adv. 2026 Jun 10;11:100591. doi: 10.1016/j.ijnsa.2026.100591 (PMC13320447; doi:10.1016/j.ijnsa.2026.100591)
Supplement: Supplementary file 3 [file mmc3.docx]

**Supplementary file 3. Search strings**

The following search terms were used to search four databases for title and abstract:

- PsycINFO
- Web of Science
- Cochrane
- Social Services Abstract and Proquest

1. “early interven*” OR “parent* interven*” OR “parent* program*” OR “parent* course” OR “parent* workshop*” OR “parent* train*” OR “parent* session*”

AND

1. baby OR infant* OR babies OR newborn* OR neonate* OR “transition to parenthood” OR “first time parent*” OR “becoming a parent”
   (using filters neonatal and 3-23 months yields very few results)

AND

1. “mother support group*” OR “peer counseling” OR “group intervention*” OR “group program*” OR “community-based" OR “social support” OR “group-based”

Adding in poverty terms ("Low SES" OR "teenage" OR "single parent" OR "vulnerable" OR "low income" OR "adolescent*" OR "poverty*" OR "disadvantaged") led to too few results.

For the fifth database Pubmed the following search strings were used:

“early intervention” OR “parenting intervention” OR “parenting program” OR “parenting course” OR “parenting workshop” OR “parenting training” OR “parenting session”

baby OR infant OR babies OR newborn OR neonate OR “transition to parenthood” OR “first time parent” OR “becoming a parent”

“mother support group” OR “peer counseling” OR “group intervention” OR “group program” OR community-based OR “social support” OR group-based‘.
