# Supplementary material for: The power of the group – Group-based parenting programmes for disadvantaged parents and their infants: a realist review
Source: Int J Nurs Stud Adv. 2026 Jun 10;11:100591. doi: 10.1016/j.ijnsa.2026.100591 (PMC13320447; doi:10.1016/j.ijnsa.2026.100591)
Supplement: Supplementary file 4 [file mmc4.docx]

**Supplementary file 4. Rating criteria**

Relevant articles were selected and rated for richness. In realist review, relevance is defined as containing information on context, mechanism and/or outcome. In this paper, relevance was defined as a document's capacity to inform the programme theory rather than its study design or outcome strength, in line with realist reviews. (Dada et al., 2023)

Articles that were deemed relevant, were then rated for richness, considering how much information they contained about the most important mechanisms of change, coming from the initial programme theory. This list is not exhaustive, other codes were identified during the coding process. **This list represents illustrative examples from the initial programme theory and did not constrain identification of additional or emergent mechanisms:**

- lifting barriers to joining: stigma/ judgment, lack of transportation or childcare facilities
- improving support network
- learning from peers
- group leader as a role model and attachment figure
- removing stress caused by financial problems and uncertainty about raising a baby
- improve parenting confidence by learning about and playing with baby
- babywearing and physical touch for intimacy and fun

1 Articles were considered ‘rich’ **** when the main outcomes contained much valuable data on the mechanisms of change, for example by interviewing participants and group leaders about their experiences with, and thoughts about, the program.

2 Articles were considered ‘medium rich’ ***

- when they contained some data on these mechanisms, mostly in the introduction and discussion or
- when they contained much valuable data on context and outcomes or
- when they contained limited data, but the data was easy to extract and could add weight to findings.

3 Articles were considered ‘a little rich’ ** when they contained some data on context and/or outcomes.

4 Articles were not considered rich * when they contain limited or no information on either mechanism, context or outcomes.

**Bibliography**

Dada, S., Dalkin, S., Gilmore, B., Hunter, R., & Mukumbang, F. C. (2023). Applying and reporting relevance, richness and rigour in realist evidence appraisals: Advancing key concepts in realist reviews. *Research Synthesis Methods*, *14*(3), 504–514. https://doi.org/10.1002/jrsm.1630
