# Supplementary material for: The power of the group – Group-based parenting programmes for disadvantaged parents and their infants: a realist review
Source: Int J Nurs Stud Adv. 2026 Jun 10;11:100591. doi: 10.1016/j.ijnsa.2026.100591 (PMC13320447; doi:10.1016/j.ijnsa.2026.100591)
Supplement: Supplementary file 5 [file mmc5.docx]

**Supplementary file 5. Coding framework**

1. *Recruitment/ Joining*

Problems, feeling bad, lonely, isolation, mental health/ financial problems, depression, low mood, high stress levels, challenges about adapting to parenthood, not coping, single parenthood, stigma (young parenthood, poverty, abuse)

Interested in learning about parenthood, interacting with other parents and babies meeting other babies

Referral by health care professional (midwife, general practitioner, social or youth care workers) or positive stories from other participants, or: being forced to join, court order, threat of child removal, child protection services

Barriers, such as transportation, no money, no childcare, no support (or even being prohibited) by family members, busy/ other obligations/ competing demands

(Not) being addressed, intervention aimed at mothers, beliefs about gender roles in parenting, (white/black parents?), grandparent

(Overcoming) distrust, negative experiences with care providers, feeling pressured, fear of judgment/ stigma, actively avoiding help and support, re-engaging with services

Miscellaneous joining

1. *Facilitator role*

Interpersonal skills, creating a safe space, key to feeling welcome and safe in the group, empathy, warmth, non-judgmental, trusting relationship, attachment figure

Personalized, out of the box, not protocolized: she came immediately, when others just made me fill out forms. investing in relationship through one-on-one care, made me feel seen as a person

Building a bridge to other help, translate for parent, re-engaging with services, improving relationships with other healthcare services, decreasing involvement of child protection services *(overlap with code cooperation)*

Reducing gradient – not too different from me, shares personal story, knows what it’s like, using humor, not authoritative, down to earth

Miscellaneous facilitator role

1. *Group*

Entry in the group, feeling awkward, nervous, scared of the group, being shy/ introvert, social anxiety, intimidated by group dynamic, don’t want to open up, rules, working together agreement/ contract, icebreaker games, welcome

Welcoming group, non-judgmental atmosphere, there is no stigma here, feeling relaxed / comfy

Identification with peers, sharing the same problems, meeting people that are *like* me, people who understand the bad stuff I went through, same gender / culture

Sense of community, improving social support, meeting other parents, making *friends*, not feeling alone, becoming a family, bonding, calling them when I have a problem, giving and receiving practical help (i.e. babysitting), accepting help and support

Learning from other parents, sharing parenting advice and experiences, who I want to be as a parent, hearing other parent’s values, comparing baby’s behavior, asking questions about baby

Eating together, cooking, makes me feel at home, informal, opens new ways of communicating, I make appointments for meeting mothers outside of the group

The program, speakers, incursions/ excursions, co-production of the program, I like that we think of something and then we can talk about it, room for individual concerns/ individual counseling when needed

Miscellaneous group

1. *Growing*

Reflection on being parented (process difficult memories), and on parenting my baby, breaking the cycle, therapeutic

Growing as a person, opening up, (re)gaining confidence, feeling empowered to learn new skills

Feeling better, mental health, wellbeing, learning emotion regulation, coping skills (mental and financial), stress reduction

Miscellaneous growing (psycho-education on mental health)

1. *(Reshaping) interaction with baby*

Parenting confidence, I am a good parent, I can do this, I know how my baby ‘works’, I know that I am important and I am doing well/ my best, reducing parenting stress

Connection with baby, mindful and playful, enjoyment, love, intimacy, improved relationship, babywearing, massage, attachment

Mentalizing, understanding what my baby is thinking, learning to pick up her cues, maternal reflective functioning (*overlap with code reflection)*

Learning about parenting, learning skills, not losing my rag, learning about developmental stages, positive communication, descriptive talking and playing with baby, that parenting has a huge impact on baby

Baby’s development, child outcomes, improving socio-emotional and cognitive development, seeing baby grow and learn, develop, less clingy, socialize with other children, being proud of baby

Sharing parenting problems, seeing other parents have the same problems

Miscellaneous interaction

1. *The future/ after the group*

Fears about future about being without support of the group, wanting to learn more, desire for the group to continue

Hope for future, motivation, seeing a future, starting work or study, feeling empowered, keeping the friendships or looking for other support groups i.e. toddler play group

1. *Professional perspective*

Training and education, social care or health care workers, experience in group work, lot of responsibility, need for super- and intervision, evaluating, learning and improving together, holding space of facilitator, nested model: facilitator ‘holds’ mother ‘holds’ baby, who ‘holds’ them?, two facilitators?

Improved way of working, changing the way we work, influencing our work with parents outside the group, rewarding, feeling good because of reducing inequality and improving baby’s chances in life

Cooperation with other organizations, making connections, time to implement, more efficient, help parents/ babies better/ quicker

Organizational context implementation, support, recruiting and retaining parents is very time intensive, need support of colleagues/ organization, workload additional to regular work, workload/ burden

Miscellaneous professional perspective

1. Miscellaneous other

**In total: 38 codes**
