# Supplementary material for: The power of the group – Group-based parenting programmes for disadvantaged parents and their infants: a realist review
Source: Int J Nurs Stud Adv. 2026 Jun 10;11:100591. doi: 10.1016/j.ijnsa.2026.100591 (PMC13320447; doi:10.1016/j.ijnsa.2026.100591)
Supplement: Supplementary file 6 [file mmc6.docx]

**Supplementary file 6. Table of included literature**

A list of abbreviations can be found at the bottom of the table.

**1 Mellow babies**

| **Author/ year** | **Article type** | **Results (taken from abstract)** | **Participants** |
| --- | --- | --- | --- |
| Puckering 2010 | Waitlist-control | EPDS score dropped from 19 to 12 (significant), while the control group showed slight increase.  Observed positive parent-baby interaction showed increase, control group a decrease. Negative interactions stayed the same, but control group increased steeply. Positive experience of mothers. | N= 11 depressed mothers  N= 6 controls (wait list) |
| Penehira 2012 | Qualitative, interview and focus groups | Increased social support. Connection formation. Reflecting on own parenting skills, increased knowledge on parenting, and feeling empowered to learn new skills. | 2 focus groups parents (N=not reported)  N=1 (interview parent)  N=2 (interview facilitator) |
| Doherty 2012 | Poster on a waitlist-control study + follow-up (based on dissertation Doherty 2019, which could not be located) | Significant increase in wellbeing. Ability to cope with parenting role and children’s behaviours, feelings of self-esteem and adequacy. Reductions in unwanted problematic behaviours from their children, increase in children’s social skills and greater movement from the clinical range to the non-clinical range for mean child behaviour scores on all measures.  Qualitative data showed extremely positive responses to the programme resources, content and process. | N= 33 fathers (children 0-5 years)  N=13 controls (waitlist) |
| Scourfield 2014 | Interviews, documents, observation | Increased parenting skills. Improved attachment. Increased social support. Increased practical skills in parenting. | N = 6 fathers (children 0-5 years)  N = 6 staff members |
| Macbeth 2015 | Systematic review and meta-analysis | Mellow Parenting has medium effects on maternal well-being and child problems and good retention rates, but: small sizes and methodological problems. | 8 papers including 9 databases |
| Scourfield 2016 | Interviews, documents, observation (extended version of 2014) | Increased parenting skills. Improved attachment. Increased social support. Increased practical skills in parenting. | N = 6 fathers (children 0-5 years)  N = 6 staff members |
| Levi 2019 | Pre <-> post intervention | Improved mental health, parenting confidence and baby conduct problems, but NOT parenting daily stress or overall child difficulties (but these were already quite low). | N=183 |
| Raouna 2021 | Pre <-> post intervention | High retention rates and satisfaction. Improvements in anxiety, overall wellbeing, parenting confidence and perceived closeness. Decrease in involvement of child protection services. No significant improvements in depression and child outcomes (possibly need longer to emerge?). Results the same for fathers and mothers. | N=70 mothers  N=21 fathers |
| Davidson 2023 | Interviews pre <-> post intervention | Mechanisms that enabled positive change:   - sense of community cultivated within group - formulating and re-conceptualizing difficulties - opportunity to reshape interactions   Beneficial components:   - facilitators’ interpersonal skills - multi-dimensional, group-based approach | N= 50 mothers  N=18 fathers |
| Davidson 2023 | Poster presentation | See above (summary of article) |  |
| Duncan 2024 | Tajikistan Quantitative pre-post intervention | Improvements in maternal mental health, parenting stress, quality of life and child behavior, lower depression anxiety (all self-report).  attendance high with 84% attending all 14 sessions. | N = 195 mothers |
| Puckering 2018 | Book chapter | Description of different Mellow Programmes and their theoretical underpinnings, and some case studies as examples. |  |

**2 Parent Infant Programme (Formerly: Up to 2 Parent and Baby Programme)**

| **Author/ year** | **Article type** | **Results (taken from abstract)** | **Participants** |
| --- | --- | --- | --- |
| Mc Gilloway 2012 | Study report of test phase | Reductions in child conduct disorder and hyperactive behavior. Improvement in prosocial behavior. Beneficial effect on parental well-being and psychosocial functioning. Improved parenting skills, more positive parenting strategies, and closer parent-child relationships. | N = 87 parents of children (3-7 years) |
| Leckey 2019 | Interviews + six focus groups | Gaining knowledge (understanding of infants' behaviors and needs, awareness of infant development, importance of interaction) and new skills. Alleviating stress and concerns associated with motherhood. Social and moral support received in the group was seen as essential for reducing social isolation and increasing confidence. However, parental attendance was a challenge, and participation tended to decline over the cycle of programme delivery. | N=22 mothers  N = 8 facilitators  N = 17 facilitators (in focus group) |
| McGilloway 2019 | Report on results of non-randomised controlled trial | Improved parenting confidence (PSOC score), also at 2-year follow-up. Improvements in child emotional support and cognitive stimulation at 8 months, but not 2 years.  No improvements in wellbeing, or parenting stress.  Lower number of infant visits to GP. More reading to infants. | N=106 (39 high-risk)  N=84 (34 high-risk) controls (care as usual) |
| McGilloway 2020 | Interviews and focus group | High satisfaction of both parents and program deliverers. Parents report a sense of community and learning. Deliverers report improved parenting capacity and bonding/ interaction. Description of implementation successes and challenges. | N=22 parents  N= 6 parents (in focus group)  N=22 implementers  N= 18 implementers (in focus group) |
| ENRICH flyer | Overview of key findings | No extra information to McGilloway 2019 and 2020.  Parenting self-efficacy and parental confidence, parenting knowledge and skills. Sensitive and supportive parenting. | N=400 |
| Hickey 2020 | Non-randomized controlled trial (underlying to McGilloway 2019) | See McGilloway 2019  NB Improvement in parenting self-efficacy was only found in the per protocol analysis. | N=106 (39 high-risk)  N=84 (34 high-risk) controls (care as usual) |
| Hickey 2021 | Interviews, focus groups,  document search | Promoting factors to implementation success:   - programme characteristics - stakeholder attitudes - -organisational and systems factors (e.g. leadership and collaboration).   Challenges:   - engagement and adoption barriers: only 12 (55%) low income mothers participated | N=22 parents  N= 6 parents (in focus group)  N=22 implementers  N= 18 implementers (in focus group) |
| Hickey 2023 | Interviews for definition of programme theory | Identification of patterns of interaction between programme, stakeholders, and context in which program is delivered, and the extent to which these interactions shape implementation and impact. | N=19 stakeholders (not parents) |
| Hickey 2023 | Book chapter | Greater sense of parenting efficacy and positive long-run changes in parents' beliefs in their ability to cope with parenthood. Improved parenting knowledge. Alleviated concerns and stresses. Increased social and moral support. | N = 212 parents  N = 168 parents controls (care as usual) |
| Hickey 2024 | Interviews and participation data. | First-time mothers are more likely to enroll than younger or lone parents. However, older age and married/cohabiting status were the strongest predictors of attending at least one-third of sessions. Qualitative findings highlighted the importance of relationship building and connection in supporting participant recruitment and engagement. Practical and psychological barriers to participation are also described. | N=106 |

**3 Mother-Baby Nurture Program**

| **Author/ year** | **Article type** | **Results (taken from abstract)** | **Participants** |
| --- | --- | --- | --- |
| Cooke 2023 | Programme description | Description of programme and of facilitator – (attachment figure for mothers) and mother roles and experiences. | N= 21 parents  N = 5 facilitators |
| Cooke 2023 | Pre <-> post intervention, no control group | Contains two studies:   1. Improvements in depression, anxiety, parenting confidence. 2. Improvements in parental reflective functioning, parenting stress, but observed interaction (parental reflective functioning) failed to reach significance. | 1. N=69 mothers  2. N=27 mothers |

**4 Young Parent Program**

| **Author/ year** | **Article type** | **Results (taken from abstract)** | **Participants** |
| --- | --- | --- | --- |
| Strange 2019 | Thematic analysis; interviews with parents and facilitators | Young parents engage in early parenting services that are welcoming, nonjudgmental and holistic. Improved parenting skills, - knowledge, and – confidence. Parents more tuned into their infants’ needs. Empowered to co-design program activities to meet their parenting and non-parenting needs. Friendships and a social support network in their local community. Linkage to community services and resources. Children cared for and stimulated with age-appropriate interactions and play. | N=20 parents  N=5 facilitators (focus group) |

**5 Baby and Us (part of Empowering Parents, Empowering Communities)**

| **Author/ year** | **Article type** | **Results (taken from abstract)** | **Participants** |
| --- | --- | --- | --- |
| Thomson 2015 | Qualitative study semi-structured interviews | Peer facilitators described positive changes in their own families, confidence, and social status. Peer facilitators’ enthusiasm, openness and mutual identification with families seen as critical to effectiveness and sustainability. | N = 14 peer facilitators |
| Harwood 2022 | Scientific article, quasi-experimental | Recruiting parents from disadvantaged backgrounds feasible. Programme completion high. Parent goals closely matched aims of programme. Parent satisfaction high. Increased mental wellbeing. Increased confidence, parenting skills and self-efficacy. Goal attainment. | N=158 parents (infants 0-12 months) |
| EPEC 2024 | Webpage NHS Scotland on experience in Sheffield | Program description. Reduction in children’s behavioural problems. Volunteer peer parent facilitators improve retention rates in groups that may not access professional support easily. Benefits for volunteers: life changing, improving confidence, qualifications and employability. |  |

List of abbreviations:

ENRICH: EvaluatioN of wRaparound in Ireland for CHildren and families. A 5-year multi-component research programme.

EPEC: Empowering Parents, Empowering Communities. A parent programme in the UK, to which ‘Baby and Us’ is a sub-programme.

NHS: National health Services, the ;ublicly funded health care system in the UK.
