# Supplementary material for: The power of the group – Group-based parenting programmes for disadvantaged parents and their infants: a realist review
Source: Int J Nurs Stud Adv. 2026 Jun 10;11:100591. doi: 10.1016/j.ijnsa.2026.100591 (PMC13320447; doi:10.1016/j.ijnsa.2026.100591)
Supplement: Supplementary file 7 [file mmc7.docx]

**Supplementary file 7. CMOC’s underlying the 6 program theories, and all quotes that they were based on**

1. **Five CMOC’s belonging to the Program Theory on ‘developing a trusting relationship’**

**CMOC 1 Mistrust and stigma**

When people in disadvantaged/ vulnerable circumstances become parents, a lack of financial and social resources, and a history of isolation and unsafe relationships, can lead to feelings of stress and overwhelm, making parenting more difficult (C), but admitting to needing or wanting parenting support (M) is hindered by shame, stigma, fear of being judged and suspicion of service providers (M), creating a (sometimes large) barrier towards joining a group-based parenting intervention (O).

| **Mellow Babies**  Davidson et al., 2023 | Feelings of stigmatization or guilt appear to disproportionately affect at-risk parents who have to navigate parenthood challenges within the realm of stressful life circumstances, further discouraging them from engaging in parenting interventions due to their fear of being judged […] most parents reported unhappiness due to isolation (n = 32), mental health difficulties (n = 35), or challenges adapting to the parental role (n = 20) before starting the intervention, “*I suffer with depression and anxiety and as a mother it did get a bit much for me, I wasn’t coping with life and being a mum and just everything*” [Mum 21]. These consistent reports of low mood, high-stress levels and social disconnection were shared equally by mothers and fathers. A few parents also reflected on the negative impact that their wellbeing and functioning had on the relationship with their baby and their socio-emotional development (n = 4), *“I was never out, I was always in the house with the wee boy. He wasn’t getting to learn anything” [Mum 18].* |
| --- | --- |
| Scourfield, 2014 | Mellow Parenting is a parenting programme for families where the children (0-5 years) are either on the child protection register or the extent and nature of associated risk factors for child development give significant concern that child protection might become an issue […] 25% of participating families had a child on the Child Protection Register […]Seventy four percent of mothers entering the Mellow Parenting programme reported at least one hostile or indifferent parent figure in their childhood and sixty percent reported no current confiding friend or family member was available for them. |
| Macbeth 15 | Our findings support the evidence from single case and narrative reviews of Mellow Parenting that a group based, attachment-informed intervention can be effectively targeted towards parent–child dyads at risk of serious adverse outcomes resulting from parental difficulties. The baseline samples for all studies included in the meta-analysis had multiple indicators for developmental risk (including social adversity, exposure to interpersonal violence, parental substance misuse, parental mental illness, or previous statutory social-service involvement) |
| Scourfield 2016 | The target fathers for ‘Mellow’ intervention are, according to Puckering, ‘vulnerable’, often with complex and multiple problems including substance misuse, mental health problems and domestic violence, as well as typically unemployment, financial difficulties, offending behaviour, poor education and poor literacy. The targeting on ‘vulnerability’ is no different from Mellow Parenting for mothers, but the issues presenting will undoubtedly be gendered, for example with men more likely to be perpetrators of domestic violence and more likely to have criminal justice involvement |
| davidson, 2023 | Pre-intervention difficulties contributed to negative feelings about attending the group. While a few parents were eager (n = 3), the majority reported feeling nervous and having low expectations (n = 54). Fathers expressed hesitation about opening up to strangers and feeling pressured by external services to attend (e.g., child protective services), while mothers’ [expectation of joining the group] was mostly characterized by social anxiety and fear of judgment of their parenting abilities. However, after attending the MB group, most participants commended the relaxed and welcoming environment of the program; a surprising element for some parents who expected to be “reprimanded” or “talked at”. |
| Davidson et al. 2023 2006; Markin | [..] it is also possible that parental attachment style may have contributed to the pre and post-intervention experiences and feelings expressed by parents. Research suggests that rates of insecure attachment styles are higher amongst at-risk individuals (Thomson & Jaque, 2017), who may display higher levels of anxiety and be more fearful of new experiences (Goleman, 2006; Markin) |
| Mellow Programmes ch 20 | Parents with LDs [learning disabilities] are a particularly vulnerable group. […] About 50 percent of these parents have their children removed largely on the presumption of incompetence, a deficiency perspective focussing on what the parent cannot do and a discriminatory practice which undermines rather than supports coping  […] The lives of the [LD] families were exceptionally complex, with partners and extended family taking an unusually active role, for protective or less noble reasons. Struggles with social services, child protection, housing and legal services were also commonplace […] Eighteen of the 24 mothers who completed the pilot programmes consented to take part in the evaluation. The mothers had very complicated life circumstances: two of the mothers grew up in local authority care themselves, six of the mothers had had previous children removed from their care and ten of the unborn children or babies were subject to child protection plans and/or their grandparents had parental responsibility. […]. There were a range of current issues in the mothers’ lives such as use of alcohol, depression and self-harm  […] Mellow programmes deliver some very subtle messages in apparently simple and concrete ways, to parents who find routine services hard to use, for fear of stigma and suspicion of service providers. A commitment to work with the most vulnerable groups in society and reduce social and health inequalities drives all the programmes. |
| **Parent Infant Program**  ENRICH- Summary | ‘harder to reach’ parents were less likely to attend the program and attitudinal and practical barriers to participation were common. [public health nurse]: “*As I say: certain vulnerable clients, to certain non-nationals, non-Irish people…You know, it can be hard to get a mixed profile really*.”  Pre-programme, qualitative findings highlighted mothers’ feelings of exhaustion and anxiety around caring for their baby. […] The results from our qualitative interviews revealed that many felt ill-prepared for parenting. Feelings of inadequacy, doubt, anxiety and worry, as well as exhaustion and isolation, were common |
| Hickey 2023 | The programme addresses psychological or structural barriers to attendance (such as fear, stigma or childcare/transport issues) and aims to build trustworthy relationships with service providers in order to facilitate a smooth transition to the IY parenting programme. |
| Hickey et al., 2024 | […] young parents, low-income families and parents from low-education backgrounds are less likely to attend parenting support (Hackworth et al., 2018; Knox and Burkhart, 2014). First-time parents may feel less confident in their parenting role and may be more likely, therefore, to enroll in an early parenting programme, although they may also experience more psychological distress that may, in turn, impact participation (Muscat et al., 2014)  […] The recruitment of parents to the PIN programme was experienced as an intensive process from a programme provider perspective. Facilitators, for example, described investing considerable time and energy into advertising and encouraging engagement at a community level. Despite this, promoting uptake amongst parents was seen as a challenge. More vulnerable, disadvantaged and younger parents were identified by stakeholders as more difficult to engage |
| **Young Parent Program**  Strange et al., 2019 | A non-judgmental environment was perceived to be very important. For example, parents reported ‘fearing judgement’ or being judged by older parents in the community and work place as confirmed by this parent ‘*A neighbour goes… like you shouldn’t have kids at your age…you’re a child yourself…why should you have kids?*  […] Furthermore young parents reported they avoided mainstream parenting groups where they felt judged and isolated. Our finding is congruent with McArthur and Winkworth (27) who found stigma both perceived and predicted resulted in young parents avoidance of mainstream services. One young parent described the welcome at YPP compared to the isolation felt in other playgroups. ‘*So I’ve tried a few different ones but it never felt right…it didn’t feel as welcoming. I just didn’t connect with the facilitators and I didn’t connect with the parents that came, I just felt isolated whereas here… I’ve never experienced those feelings.’ (P9 child 30 months*) |
| Strange et al., 2019 | Half of the parents stated they were isolated prior to joining the program and many of those isolated had not known other parents prior to joining YPP. Some parents talked about how becoming a parent resulted in losing old friends who did not have children […]. Others had few friends before making friends through YPP: ‘*I felt really alone before I started coming ‘cause I didn’t know where else to go… and I didn’t really have any friends*.’ |
| Strange et al., 2019 | Many of the parents in our study had complex social needs, as found in other studies with young parents (11). Complex social needs along with stigma have been identified by McArthur and Winkworth (29) as factors that prevented the realisation of the ‘hopes and dreams’ of young parents. |
| **Mother Baby Nurture**  Cooke et al., 2023 | As a requirement of acceptance into the MBN program, the dyad had been assessed as “vulnerable” in some way (recently discharged from mother–baby psychiatric unit, screening by a GP or Child Health Nurse indicating elevated symptoms of distress, referral from a mental health provider), yet not “high risk” (active psychosis, addiction, family violence or department for child protection involvement). These mothers may present with varied symptoms including but not limited to; history of pregnancy/birth trauma; loss; family/relationship breakdown; anxiety and/or depression; feeling overwhelmed or emotionally detached towards baby/loved ones. Mothers may also report concerns about their baby’s crying, feeding, sleeping habits or developmental milestones.  Pre-test Parental Distress scores indicated that many mothers commenced the group in a distressed state, struggling with internal adjustment to the parenting role; a known correlate to parenting dysfunction The heightened distress experienced by some mothers is likely to be related to experiences of early attachment relationships and insecurities activated by the presence of the infant, leading to a high level of unexplained arousal, accompanying strong feelings of fear of abandonment or a sense of overwhelm. |
|  | the pre-test score of 3.91 suggests that PRF was limited for most participants, which was likely to have detrimental impacts on the mothers’ capacity to provide sensitive responsive care for her infants |
| **Baby and Us**  Thomson et al., 2015 | Peer leaders [are] assumed to reduce initial barriers to help-seeking, particularly those related to socio-economic status, cultural diversity, stigma and distrust of professionals. |
| **Harwoord 22** | Baby and Us programmes were largely located in highly disadvantaged communities – over 70% were in the bottom third most deprived areas, characterised by low employment, high crime, low education, poor housing quality and low incomes  Families were eligible for inclusion if a primary parental caregiver (“parent”) had self-identified difficulties in managing their infant, aged between 0–12 months, including parental stress, parent-infant relationship difficulties, interaction difficulties, and social isolation. The inclusion criteria was outlined using parent friendly language on all recruitment information |

**CMOC 2 Developing relations**

When parents have little trust in services (C) and organisations prioritise pre-engagement communication, involving personal contact and/or the use of intermediaries that parents know and trust (Mresource), then parents are more likely to agree to attend a parenting intervention (O) as they begin to develop trust (M resp) in the intervention representative, feel less judged (Mresp) and less threatened about the consequences of attending a programme (e.g., the worry that their child will be removed from their care is reduced) (Mresp).

**CMOC 2a Hiding**

When parents do not trust intermediaries or feel pressured (M), they may not want to join or may even actively hide their circumstances and problems (O) (e.g., learning disabilities, domestic violence) (C)

| **Mellow Babies**  McGilloway, 2012 | Parents believed that the future implementation of the programme could be enhanced through improving early referral systems. For example, it was suggested that more public health nurses, pre-schools and national schools could be informed about the programme |
| --- | --- |
| Mellow programs CH 20 | The first contact is by a letter, quickly followed by a telephone call, as literacy can be a hurdle. The first meeting is always in the parent’s home, or in a place the parent nominates. The programme is presented as an offer, for them to accept or decline. Even if there is an external mandate, the wishes of the parent are given precedence, with the Mellow Parenting practitioner taking responsibility for reporting back to the referrer that the group is not the right intervention for this parent at this time. Under careful negotiation, parents who previously felt they had few choices may choose to join the group |
| Hickey et al., 2023 | Fathers expressed hesitation about opening up to strangers and feeling pressured by external services to attend (e.g., child protective services) |
| Puckering, 2008/2009 | Despite much liaison with maternity services, few women with LDs [learning diabilities] were referred in pregnancy. Mothers-to-be were understandably reluctant to reveal a vulnerability they knew was associated with a high risk of children being removed. Busy midwives were perhaps unaware of the woman’s difficulties or reluctant to probe for fear of stigmatising |
| Davidson 2023 | Fathers reported feeling “seen and heard” within the MB group, disrupting previously reported narratives of fathers feeling like the “invisible parent” by healthcare professionals and services during the perinatal period |
| **Parent Infant Program**  Leckey et al., 2019 | recommendations by community organisations and PHNs to attend the programme, were viewed as having positively influenced recruitment and minimised drop-out. For example, a large number of mothers who participated in the programme did so on a recommendation from their PHN or family resource/local health centre. Thus, the involvement of community healthcare providers/practitioners facilitated the recruitment and engagement of more vulnerable or disadvantaged participants and indeed, parent reports suggest good working relationships with facilitators throughout the programme.  [...] The findings from the facilitator interviews also demonstrated the importance of establishing an informal relationship with mothers prior to programme commencement to maintain engagement and reduce drop-out. |
| **Hickey et al** | *“When we [facilitators] were doing all the ringing [for recruitment], even though it took ages, people bought into it because they knew who was doing it – they liked the sound of your voice or you made a connection with them*.” |
| **YPP**  **Strange 2019** | Referral to YPP was commonly through services such as parenting support organisations, child health nurses, antenatal general practitioner clinic, as well as self-referral through social networks. |
| **Baby And Us**  NHS Scotland Early Intervention Framework webpage | [program trainer] explained how recruiting for the group is done by the parent group leaders going into their schools, speaking to other parents in the playground or community who they have a connection with.[…] [program trainer] also feels another barrier to recruitment can be the stigma associated with parenting in Britain. However, having other parent volunteers is *“really helpful and normalising”* and the key to promoting a community network is relationships. |
| same | [program trainer] explained the challenges of going into communities where you do not speak the language and are not part of the community: “*When we've got a parent group leader in post he was able to say that you're not advertising it in the right way…the same with the Chinese community. […] they've been able to promote on Wechat to their Chinese community […], whereas again we would try and do that but it just wouldn’t translate as well... if it's an actual Chinese parent that's talking then they're much more receptive really*.” […] Also, having a male worker, who is Slovakian, a single dad, has allowed successful delivery of BAP to the Roma community which had not been possible by staff. The dad knows the community well, so was able to recruit and retain a group of Roma families to stick with the programme which had not been possible when run by staff. |

**CMOC 3 Tailoring**

When the parenting program addresses the specific needs of disadvantaged parents (C) by tailoring the program -and the communication about it-, to parents’ language levels and to their need for social interaction and network, self-care and improved mental health, and parenting tools and knowledge (M), chances of them joining are bigger (O).

| Hicky 2019 | Initial decisions to participate may be positively influenced by a perceived need and desire “to be a better parent”. |
| --- | --- |
| Hickey 2024 | personalising communication strategies may be needed to ensure that programmes are appealing to all parents. For example, communications that emphasise skill development and support during the transition to parenthood may be an effective recruitment strategy for first-time parents; however, parents of additional children may be more attracted to other elements of these kinds of programmes, such as network building with other parents. |
| Davidson, | There is inevitably some tailoring of material [for father instead of mothers]. The sessions on ‘you and your body’ and ‘pregnancy and birth’ were clearly different in content from the equivalent sessions for mothers. Interestingly, the session on self-esteem from the mothers' programme has been replaced with ‘being a Dad’. |
| MELLOW PROGRAMMES | In 2012, Mellow Parenting and Mencap developed and field tested a programme to support parents with LD from pregnancy to age 1. We had the support of the parent’s reference group from the Elfrida Society, an advocacy service for people with learning disabilities and difficulties, which vetted our materials for their appropriate use of language and accessibility. |
| MBN  Cooke | Suggestions [by the parents] for improvement included […] longer sessions combined with an extended course; greater focus on self-care, gratitude and mindful ness; creating more space for mothers to interact with each other |
| Baby and Us Harwood | The most common [goal of the parents] being to gain knowledge about general parenting (n=30, 21.9%), improving knowledge about routines e.g. feeding/sleeping (n=23, 16.8%) and increasing parent’s social network (n=20, 14.6%). Fifteen parents (10.9%) stated their primary goal as improving their own mental health, which was not directly addressed as a programme aim. |

**CMOC 4 Unburdening parents**

When organisations provide the parenting group with facilities such as transport, meals, childcare (and/ or allowing the infant into the group) and low-literacy or translated materials (Mresource), parents who lack social and material resources (C) feel unburdened, (Mresp) and nurtured, (Mresp) increasing their developing trust (Mresp) in the intervention and lifting practical and financial barriers (Mresource) to joining (O). Where such facilities are not provided as much (PIN) (Mresource), it may be more difficult to engage more vulnerable parents (O).

| **Mellow Babies**  Raouna, 2021 | One of the strengths of MB that differentiates it from similar early intervention parenting programs is the provision of transport, childcare, low-literacy materials, and daily meals to all parent-baby dyads enrolled in the groups, which can positively contribute to overcoming attendance and commitment barriers, usually encountered by ‘at-risk’ parents |
| --- | --- |
| Puckering, 2010 | Active care was taken, in facilitation, to provide an experience of nurture and acceptance for mothers – beginning to meet some of their needs that so often get overlooked when caught up with caring for a new infant and wider stress. Thus providing the basics: a safe environment, transport, lunch and, of course, respect/valuing/ empathy, were important containing components. In turn mothers became more equipped to provide this care for their infants. |
| Scourfield et al., 2016 | The intervention also models ‘wrap-around care’, with fathers transported to and from the venue and children looked after in a crèche during the group sessions. |
| MELLOW PROGRAMMES | To ensure that the most vulnerable families, who are least likely to access the very services they need (Maybelis & Maryatt, 2011), are recruited and engaged, transport assistance and light refreshments are provided, to make parents-to-be feel welcome. |
| **Parent Infant Program**  **Hickey 20** | infants included in the current study were significantly younger than those recruited by Jones and colleagues (average age in weeks = 12.27) and it is possible that parents of younger infants found it more difficult to participate in the group-based intervention. Thus, early intervention and prevention-oriented supports delivered at the very earliest stages of parenthood, may require particular attention to ease of access. Additional supports for parents (e.g. travel supports) to promote engagement may be needed, whilst the quality of facilities available to parents upon attendance (e.g. accessibility of venue with pram, baby changing facilities), should also be considered. |
| **Hickey 21** | Parents’ inability to attend due to lack of transport, absence of childcare, return to work, and/or due to lack of childcare supports were identified as barriers to attendance |
| **Enrich ,** | **m**ore vulnerable families attended significantly fewer sessions |
| **ENRICH 2** | A number of parents also commented on their inability to attend the IYPTP programme, as toddlers could not attend and childcare costs were prohibitive for some |
| **Young Parent Program**  Strange et al., 2019 | The program is provided in partnership with a locally placed early years’ service that offers intensive family support, including advocacy, home visits and transport if needed. [...] The program includes transport to the program and assistance with other community services as needed |
| **Mother Baby Nurture**  Cooke et al., 2023 | Whilst many therapeutic services are reliant on childcare and both the mother and child’s willingness to accept alternate care, MBN includes the infant in the group, enabling the very young (often breastfed) infant to participate |

**CMOC 5 Initial trust**

When parents that are prone to feel judged or stigmatized, initially join groups (C) where the group facilitators are relatable and use a non-judgmental, open, and empathic approach (M resource), then parents will feel welcome (O) and begin to lower their defenses (O), due to the development of initial trust in provider and hope in what the program can do for them (M resp).

| **Mellow Babies**  Puckering, 2008/2009 | The strength of the intervention lies with the practitioners, who must build a trusting relationship with parents who have little experience of trust in their lives, starting from childhood through to current partners and services. |
| --- | --- |
| Davidson 23 | Additionally, gender-specific groups appear to have been especially important for fathers, which is in line with findings suggesting that mixed-gender groups can feel unwelcoming and intimidating to partners (Smokowski et al., 2018; Panter-Brick et al., 2014). |
| Penehira 12 | *They treated you like a mother not somebody that is just at home and you are slaving cleaning the house all day and washing dishes is all your life and things like that.* |
| Mellow programs CH 20 | Given that all the parents who attend do so because they see some hope for themselves and their children in the group, attendance is high and attrition low. |
| hickey 21 PIN | These findings are in line with the small number of previous process evaluations of parent-training programmes which show that parents’ feeling of support within the context of a group-based intervention, and perceptions of alliance with implementers, are important factors in the effectiveness of group-based parenting programmes. |
| **Young Parent Program**  Strange et al., 2019 | Facilitators provided a welcoming, non-judgemental and caring ‘wrap-around service’ where parents shared their parenting challenges. |
| **Baby and Us**  Harwood et al., 2022 | Within the targeted community, an open access approach is typically used, rather than formal referral or selection of high risk individuals. The peer-led format is associated with high levels of parent engagement, acceptability and reduced stigma. |
| Harwood et al., 2022 | B&U groups succeeded at attracting parents from minority ethnic groups at rates higher than community base rates. This is important considering the multiple barriers to engagement that parents from ethnic minorities can encounter (Forehand & Kotchick, 1996) […] Rates of English as a second language were high, highlighting the attractiveness of B&U for families who might otherwise be marginalized. |
| NHS Scotland Early Intervention Framework webpage | The parent volunteers come from various different backgrounds (e.g. Roma, Chinese, Arabic) and include some grandparents as group leaders. […] [trainers] feel that the key to EPECs success is parents talking to other parents, rather than professionals, […] which helps parents to feel supported. |

1. **three CMOC’s belonging to Programme Theory on ‘Facilitator creates safe space’**

**CMOC 6 Creating a ‘safe space’**

When parents who have experienced hardship and stigma (C) participate in a programme where group facilitators promote a supportive, non-threatening and inclusive intragroup environment or ‘safe space’, with a focus on strengths and support, explaining group rules*,* and using humor to lighten up the atmosphere (M resource), parents begin to feel welcome, valued and cared for (M resp), and start to engage with the group and the program (O), and this (though it may take time) may lead to a higher level of parent engagement and satisfaction (O).

| **Mellow Babies**  Penehira & Doherty, 2012 | Facilitators commented that the program is well structured and that they were able to create a safe environment allowing participants to share with others [..] *“And then you learn to respect the others’ lives. You end up respecting them and what they have been through.”* |
| --- | --- |
| Puckering 2010 | Mothers had also been found to be readily able to identify their failings but had found it very hard to give themselves credit for what they were doing well. An important part of the facilitators’ role was in drawing attention to mother’s success and skill |
| Scourfield, 2014 | The ‘relaxed and happy’ feel of the facilitators at the start of the day was said to be infectious: ‘*it kind of makes you change and you feel relaxed and ready for the day’ (Joe, father).”* There was surprise expressed about the fact that humour was allowed, given that an ‘intense’ environment had been expected |
| Penehira & Doherty, 2012 | Being respected and listened to, instead of constantly undervalued, as many of the participants had been, played a significant role in enabling participants to value themselves, to grow, and to be ready to learn new skills. |
| Scourfield, 2014 | John made an explicit connection between this reluctance to talk and what is typically expected of men, when he said ‘*I don’t know if it’s something to do with their manhood or whatever’ but that some men would not want to ‘talk or open up or anything’, at least for the first few weeks* |
| Davidson et al., 2023 | Specifically for parents involved with social services, MB was perceived as a space that “allowed” them to parent their child, “*I didn’t feel like I was being watched or judged. I could just be mum to her*” [..]. |
| ??YPP | During the first visit to the group the staff discuss with new parents a ‘working together agreement’ that includes respectful interactions with peers, children and staff. For example, the use of appropriate language, making new people feel welcomed, listening to others, confidentiality, and no shouting at the children. |
| **Parent and Infant Program**  Leckey et al., 2019 | All mothers praised the facilitators for their constant support and encouragement. They commended the approachability of facilitators in providing a safe and welcoming environment where everyone’s opinion was valued: The public health nurse was so nice, lovely, really welcoming ::: really happy, delighted to see us and telling us we were brilliant for getting out of the house with baby. |
| Hickey et al., 2024 | [..] facilitators’ ability to connect with parents and to create a safe space for parents to engage in group processes was viewed as critical. This included facilitators’ skill in managing the interpersonal processes within the group meetings and reducing any potential conflict, as well as a sense of intimidation: […] “*you have to be able to make a connection with the participants [...] non-threatening, non-judgemental […] as a facilitator, I would have had our ground rules [...] that “respect everybody, listen to everybody”, that “we’re all here [..] on an equal level*” |
| Hickey et al., 2021 | The personal qualities of programme providers were viewed as crucial to implementation outcomes. A warm, friendly, and non-judgemental approach and facilitators’ ability to establish supportive relationships with parents was perceived as helping to promote a supportive intragroup environment, and ultimately a high level of parent engagement and satisfaction with the programme: *“I did find [the group] supportive. […] It can be hard initially in the first few weeks because nobody knows each other ... but it was a safe group process, it was a supportive group process.”* |
| **Young Parent Program**  Strange et al., 2019 | *When you walk in you’ll have one of the girls come up to you, like the girls that work here and they'll say how are you feeling? How's this going? …they will individually check up on everyone. I think that's more than enough to know that someone actually cares.’* (P5 child 12 months) |
| Strange et al., 2019 | One facilitator described ‘picking up positives that someone is doing’.‘… *so when they see someone doing something really good… they just comment “You’re doing a really good job.” …just having that bit of positive feedback from someone who cares about them… because a lot of them don’t have that (at home).*’ |
| **Mother Baby Nurture**  Cooke et al., 2023 | By maintaining consistent, thoughtful, compassionate, and accepting stance, facilitators offer a potentially new experience for mothers where these qualities may have been longed-for but not experienced in their relationship with their own caregivers. Once experienced, these mothers may become able to draw on an internal representations of a ‘good grandmother’ facilitator to help contain and make sense of thoughts and feelings in a new and enriched way. |
| **Baby and Us**  NHS Scotland Early Intervention Framework webpage | [A peer support leader] feels that a key to the success of EPEC is the sense of inclusion and connection it gives to individuals through the community, networking and support that it provides. “Parents knowing that they [group leaders] are willing to stand up and be the spokesperson and support families, it reaches out to those people and includes them.” |

**CMOC 7 Relatability promotes safety**

When parents have developed initial trust in the group facilitator (C) and the facilitator demonstrates relatability to parents e.g., similar backgrounds, or challenges with parenting (M resource), parents are more willing to engage in group activities (O), more open to confrontation/advice about their parenting practices and more likely to take responsibility for their behavior and their communication practices with other parties involved with their children (O).This is because parents feel a shared understanding of their experiences, which makes the facilitator a relatable and credible source of support and guidance (M resp).

| **Mellow Babies**  Penehira & Doherty, 2012 | Working with the [program facilitators] was considered valuable by the participants who perceived that the facilitators have experienced for themselves what they are teaching. The validity gained with Māori women, Māori mothers teaching Māori mothers, seems a critical element underlying people’s “buy-in” to the program. […] *“it is [facilitator1] and [facilitator2] that are our backbone. They give us encouragement and guidance — they are like what ministers do in a womanly way, how we cope with our children*.”  […] *And because you are Māori, even though they are quite a bit older than us, some of the things they have been through is similar to what we have all grown up with and been through or whatever.*  *So you know it has meaning or there is truth behind it with the people that are facilitating it and they are parents as well*. |
| --- | --- |
| Davidson et al., 2023 | Two to three practitioners facilitate each group, of which at least one must match the gender of the parents in the group. |
| Davidson 23 | gender-specific groups appear to have been especially important for fathers, which is in line with findings suggesting that mixed-gender groups can feel unwelcoming and intimidating to partners (Smokowski et al., 2018; Panter-Brick et al., 2014). |
| Scourfield et al., 2016 | fathers valued the facilitators sharing their own experiences: “*They were down to earth, they felt like one of us sort of thing. They didn’t come across as too authoritative or anything like that. (Brian, father) We still talk to them like we’d talk to a pal in the street, in that context (Phil, father)”* |
| Scourfield et al., 2016 | The following instance from the group we observed illustrates the skilful handling of one case: One of the facilitators brought up the subject that this dad would need to sort out with the boy's mother how they set consistent boundaries for him. The facilitator clearly said that they thought that at present this child was at risk. The dad responded very openly to this conversation, and I do not think this would have been possible without the positive and encouraging relationship they have so far worked hard to build. |
|  | Some good quality motivational and social skills work had been undertaken by the facilitators and arguably it was attending the Mellow Dads group, with its emphasis on nurturing fathers and respecting their views, which made this work achievable. We could speculate that the fathers took advice on dealing with statutory child protection processes much better from the Mellow Dads staff, with whom they had built good rapport, than from the statutory caseworkers from social services responsible for child protection. |
| **Mother Baby Nurture**  Cooke et al., 2023 | The role of the MBN facilitators is to create an emotionally containing environment for the mothers and infants while making mentalizing explicit through maintaining a stance of curiosity and reflection. The facilitator holds the baby in mind as a separate being whose experience and behaviours are meaningful while holding the mother’s experience, even though these may seem intolerable or distorted at times [22]. To sufficiently hold the powerful projections and primitive processes in the mother-baby group, two facilitators are recommended [23]. The facilitators narrate their observations aloud in the group, at times making sense of differences of perspective, modelling not knowing the other’s mind as well as sharing co-joint intent to work together to support group members [24, 25]. There may be minor misunderstandings and differences in the facilitator’s perspectives, and these distinctions are talked about to model “good enough” parent relationship to the mothers. Facilitators may stop and rewind, slow the pace or seek clarification in an effort to reflect upon a shared moment when a member responds in a particular way. |
| **Baby and Us**  Thomson et al., 2015 | Peer leaders may have enhanced credibility for service users because of shared characteristics and experiences. […] there is potential for enhanced treatment participation and positive working relationships due to mutual identification and engendered trust. |
| Harwood 22 | The peer-led format is associated with high levels of parent engagement, acceptability and reduced stigma |
| Webpage NHS | Also, having a male worker, who is Slovakian, a single dad, has allowed successful delivery of BAP to the Roma community which had not been possible by staff. The dad knows the community well, so was able to recruit and retain a group of Roma families to stick with the programme which had not been possible when run by staff. [trainer] felt that as he was from the same background as the families, he was delivering to they could relate to him. He was also able to translate the resources for families. |

**CMOC 8 Facilitator learns too**

When new facilitators from diverse backgrounds with limited previous experience (C) engage in initial training and education and receive ongoing support and supervision (M Resource), they develop an understanding and respect for participating parents and feel more confident (M Response) to incorporate new learning into their work practices (O).

| **Mellow Babies**  Penehira & Doherty, 2012 | *“And then you learn to respect the others’ lives. You end up respecting them and what they have been through. I didn’t like all the stories that came out but it was good because they could then be supported to move forward.”* |
| --- | --- |
| **Parent Infant Program**  ENRICH-2 | Facilitators, particularly PHNs, also described how their involvement enhanced their own capacity and professional expertise. For instance, they reported integrating the principles of peer-led learning into their own clinical practice which, in turn, was seen as enriching their work practices and improving their interactions with parents more generally. |
| Hickey et al., 2023 | Training and facilitator support were understood to be critical to promoting ongoing, high-quality service delivery and, in turn, positive outcomes for programme participants […]. Ongoing peer support and coaching are also provided for […] facilitators to strengthen facilitator skills and confidence. |
| **Mother Baby Nurture**  Cooke et al., 2023 | Facilitators are recruited from different disciplines including psychology, social work, counselling, midwifery, and other relevant fields of allied health. Each facilitator has undertaken a three-day manualised MBN training process [14] and participates in ongoing monthly reflective supervision. […] Reflective supervision is a vital ingredient when working with young children and their caregivers in the shared exploration of the emotional content |

1. **two CMOC’s belonging to the Program theory on ‘Finding new family: the power of the group’**

**CMOC 9 Not being alone**

When parents with little social network and similar life experiences (C) are provided with a safe space where their experience is valued and respected, the opportunity to share experiences and challenges with other parents (M resource) provides them with feelings of connectedness and mutual support (often referred to as ‘family’) (M resp), and feelings of normalization and relief from recognizing the universality of their experiences (M resp), reducing social and emotional isolation (O),improving their mental health and parenting confidence (O), and being more open to learning (O). This connectedness and openness to learning may transfer to other (future) relationships in their lives (O).

**CMOC 9a Challenges with engaging**

When parents in a group are of different genders, educational or cultural backgrounds, are at very different stages of processing their past, or experience significant other life stressors (e.g. mental health illnesses) (C)*,* or when the group is too small (C), parents may feel disconnected from the group (M) and either drop out completely or not engage fully (O).

| **Mellow Babies**  Puckering, 2010 | Whilst initially engaging mothers in a group environment may require careful preparation, the power of the group includes reducing isolation and the direct message/experience of ‘not being alone’, clearly a component valued by the mothers in the group |
| --- | --- |
| Penehira & Doherty, 2012 | Being a valued member of the group was a significant experience for many of the women, most of whom had not experienced this in any other aspect of their lives. This enabled them to share openly and to build trust with peers and facilitators, which ultimately built a stronger learning environment providing greater impetus for change. “*And it is reassuring. You can feel isolated sometimes, really lonely and then you say “I am just going crazy.” And for me personally coming here is like time out — it is like stress relief. And then we have our [korero], our talks and it is like I just can’t wait to get here to meet with my classmates because they are like sisters now.”* |
| Penehira & Doherty, 2012 | *Culturally … we can understand where we are coming from because we are not individual based — our thing is about coming together as a Whānau [extended family] and that is where our strength is … we understand that dynamic*, |
| Davidson et al., 2023 | Parental reports […] indicated that the act of sharing experiences within a homogeneous group elicited a realization that they were not alone and reduced emotional isolation, “*you’re realizing that it’s not just you that’s been through bad stuff and sharing that with somebody, how you felt - is really comforting*” [..]. Discussion of challenging experiences within the context of the group setting was also seen as an enabler to cultivate a sense of community and belonging that was valued by the majority of parents, “*we’ve become more of a family now*” |
| Scourfield, 2014 | The fathers spoke of valuing time spent with other people who are in similar current circumstances or who have had some similar experiences in life: “*It kind of makes something click and you say to yourself, this person has been through or is going through the same as you. It might be worth having a listen and bouncing a few ideas off each other.” “After a few times, I was, like, ‘this is good’, because I actually learned things really. I'm not the only person stressed out with my life. Other people have stress”* |
| Levi, 2019 | However, it should also be noted that one mother [Mum 2] reported experiencing no change in her wellbeing. This appeared to relate to the feeling that the group was not relevant to her needs and therefore, feeling disconnected from other group participants |
| Scourfield et al., 2016 | There were instances when fathers were clearly looking for advice on aspects of parenting they found difficult, but advice-giving is not the approach of Mellow Parenting. […] Input from other group members was more difficult to generate […] because of the small size of the group. |
| Davidson et al., 2023 | Of note, two parents reported that they did not enjoy discussions around interpersonal adversity, criticizing the content and format of the group as being largely focused on past experiences and expressing a desire for a more uplifting and future-focused element to be weaved into the intervention, “it’s kind of been a negative experience because we’re just permanently going over the crap that we’ve been through in life” [Mum 2]. Such reflections pointed to the importance of group members [being] at the “same stage” of processing their experiences”. |
| Davidson et al., 2023 | However, it should be recognized that sustainable change depends upon a complex interplay between capabilities, motivations, and contextual factors (Platt & Riches, 2016), in which desire alone is insufficient (Byng-Hall, 2008). [..]. It is, therefore, important to acknowledge that although MB targets parents experiencing psychosocial difficulties, within the current sample, there was still variation in the level of higher education attained, employment status, severity of mental health issues experienced and child-rearing circumstances within the context of lone parenthood and varying custody arrangements. Such factors could have mediated the differences observed in the perception, engagement, and experience of the intervention, influenced by elements such as attendance motivators (e.g. self-referred vs. court-referred) and competing demands (Mytton et al., 2014; Whittaker & Cowley, 2012). |
| Davidson et al., 2023 | Additionally, gender-specific groups appear to have been especially important for fathers, which is in line with findings suggesting that mixed-gender groups can feel unwelcoming and intimidating to partners (Smokowski et al., 2018; Panter-Brick et al., 2014). |
| Scourfield et al., 2016 | The rationale for offering single-sex interventions was evidenced from other parenting programmes (Bakermans-Kranenburg et al., 2003), in that fathers being present can ‘dilute the effect on mums’ (Puckering, at interview). Also it is thought that some women would find the more personal discussions difficult in the presence of men if they have experienced domestic violence or sexual abuse. […] When the numbers of men attending programmes are low, practitioners may find it more difficult rigidly to apply criteria for cohesive group membership. For example, in the group we observed, the ages of children ranged from four months to nine years. This wide age range is difficult in terms of finding appropriate activities and in terms of sharing experiences across the group |
| Davidson et al., 2023 | Perceived reductions in social and emotional isolation held a significant value to the parents completing the MB program, mirroring a vital intervention outcome when engaging parents living within the context of multiple psychosocial adversities (most participants at the beginning of the program lived in highly deprived areas, and/or experienced a mental health issue, were unemployed and single parents). |
| Puckering, 2010 | mothers had found being the provider of a solution for another mother very empowering. |
| **Parent Infant Program**  Leckey et al., 2019 | All mothers who attended the programme spoke of the benefits of sharing with other mothers, both for normalising their experiences and enabling them to regain control of their lives. These new connections and interactions helped to promote confidence in their parenting techniques: “*Meeting new mums and getting information that you don’t know as a first time mother ::: because you think you’re the only one so it’s good to be able to be able to talk to people*.” |
| Hickey et al., 2024 | Connection with and between parents through […] social media groups was seen as helping to foster intragroup support and communication. Importantly, this type of communication and “check in” system was also considered to be less threatening for more vulnerable and younger service users who may feel uncomfortable with traditional “buddy calls” systems: […] *Thank God for social media – it is just fantastic [...] One of the girls was having serious problems with teething and at 4:00 in the morning, she sent out a message and [...] two of the mums were actually up at that hour of the night, and she said, “suddenly it didn’t seem as bad, I had someone to talk to.”* |
|  | Additional barriers highlighted by programme providers [..] included a lack of support and encouragement from within the family network for programme attendance. This was seen as a bigger barrier for parents from more disadvantaged, vulnerable backgrounds and facilitators felt that extended family networks may perpetuate stigma and/or negative attitudes towards parenting support.[…] Additionally, a small number of facilitators also noted that parents who lived close to their own family (e.g. mothers and sisters) may not feel it necessary to attend group-based parenting supports: |
| **Young Parent Program**  Strange et al., 2019 | The combined benefits such as adult company, advice and reassurance from participating in YPP sessions are illustrated in this quote: ‘*Being a first time mum and not having a family or friends around… it was kind of my way of reassuring myself that I’m not a bad parent... that I do know what I’m doing and my child is okay. I guess it was for a little bit of advice and reassurance and then at the same time it was talking to other human beings instead of not just taking baby talk*”  [..] A few parents had not extended their contact with other YPP parents outside the program and generally these parents had histories of mental health, bullying or trauma that made it difficult for them to get to know others as described here.  ‘*I find it really difficult to connect with people on that friendship level mainly because of my anxiety and my depression. So it’s hard to connect and find something that we’re both interested in… and I get really socially awkward when I’m talking and meeting new people*”  (Is that harder or easier talking to people now you’re a parent*?) It’s easier in some ways. I still get a bit uncomfortable and awkward but you can talk about how your child is doing… that they can crawl, or they can walk… and all the parents can relate to that but it’s still a bit uncomfortable.’ (P11 child 9 months)* |
| Strange et al., 2019 | Socialisation was important for isolated parents with poor social support, particularly for those who had current or past history of mental health problems. ‘*Socialising for me… so I don’t get really bad depression… cause I’ve had depression before*.’ Almost half of the parents [..] disclosed to the interviewer a history of mental health problems existing before pregnancy and four identified as having postnatal depression. Five parents disclosed a history of trauma or domestic violence. |
| **Mother Baby Nurture**  Cooke et al., 2023 | Mothers expressed the value of sharing and listening to other mothers’ experiences, which they described as being safe, open, relaxing, healing, comfortable and supportive.  “*A safe space to explore thoughts and feelings and reflect on the past and how that has an effect on my parenting*”. Connecting to other women was cherished, “*The ability to talk honestly and be supported and not judged*”. Other comments reinforced the importance of “feeling normal and not alone and knowing you’re not the only mum struggling”.  […] The group provides a safe container from which its members (mothers and infants) can begin to trust in the observations and feedback made by other members and become more receptive to new learnings. The experience of authenticity and openness can support the mother to develop an experiential understanding of social environments and interactions, a process defined as epistemic trust. The benefits of epistemic trust are expected to continue well after the facilitated group ends, leading to sustained supportive relationships between group members, which act as a steppingstone to wider social contexts |
| **Baby and Us**  Webpage NHS Scotland Early Intervention Framework | The opportunities to gain support from others and build connections is also a huge benefit. “*Some of the families, live in the same community and they've never met each other…They chat with each other at the school gates, become friends, get a wider circle of friendships and a support network… making new connections …within their communities* |

**CMOC 10 Learning from peers and facilitators**

When parents who are not confident in their own parenting styles, feel connected to other parents in the group (C), the group safe space, supported by the group facilitator, provides a platform for parents to observe parent-baby interactions (live or through video), discuss, share views, problem solve and learn together through giving and receiving advice or learning about parenting techniques (M Res). Parents realise that other parents have similar and valuable experiences to learn from, feel reassured and more confident in their own parenting choices (Mresp) and develop parenting knowledge and skills, next to friendships with group members (O).

| **Mellow Babies**  Puckering, 2010 | mothers had found being the provider of a solution for another mother very empowering. [..] and others [….] could be reticent about being in the spotlight, but enjoyed and learned from seeing how others coped. |
| --- | --- |
| Penehira 2012 | Interestingly there is just one word, ako, in the Māori language that is used for both teaching and learning […]  many of the participants articulated the benefits of sharing their experiences and knowledge with others, as well as learning from the experiences and knowledge of their peers. “*I feel less judgemental like around other mothers. I won’t sit there and go ‘ooohhh,’ because I just know that we all have our problems.”* |
| Scourfield et al., 2016 | *It kind of makes something click and you say to yourself, this person has been through or is going through the same as you. It might be worth having a listen and bouncing a few ideas off each other*. (Joe, father)  *After a few times, I was, like, ‘this is good’, because I actually learned things really. I'm not the only person stressed out with my life. Other people have stress in them.* (John, father)  “*Getting to watch the video back and seeing his development (…) when I first came and I was rubbing his back, I have always done that and I never knew that I done it. And people pointed it out and said it was a comfort thing. That makes you feel better about yourself* . [(Joe, father)]  There were instances when fathers were clearly looking for advice on aspects of parenting they found difficult, but advice-giving is not the approach of Mellow Parenting. […] Input from other group members was more difficult to generate […] because of the small size of the group. |
| **Parent Infant Program**  Leckey et al., 2019 | Group discussions provided a valuable opportunity to listen to others’ views, problem solve and learn about new and/or different parenting techniques: *So that was kind of nice to hear of someone who’s gone through it before. But even the other first-time mums, everyone develops at a different rate, so you know somebody might have done something or somebody would come in with a question and we’d kind of brainstorm it out.* |
| ENRICH-2 | The groups were seen as providing a source of informal knowledge and emotional and moral support, where mothers could openly exchange advice on parenting needs and express their frustration or anxiety in an informal setting. The process of peer-led learning reportedly helped to strengthen parenting confidence and empower parents to develop their own solutions to parenting challenges. The experience of interacting with other mothers and babies was highlighted by interviewees to be hugely beneficial and a significant source of knowledge and skill development as well as support and friendship.*”You just feel like you’re more plugged into a network, plugged into a community and on a very practical level, you can chat to other mums and say 'does your baby do this?'* |
| Hickey et al., 2021 | Some parents were selective in their application of the parenting techniques and information offered during the course. *[…] “In the room there’s a mixed pot of people and other people… certain things are important to them and you might feel that you don’t form the same opinion as them.[...] it’s having the confidence to figure out what type of parent you want to be.”* |
| Hickey et al., 2023 | Furthermore, it was understood to be the kind of intervention which would ultimately help to ameliorate and prevent developmental disadvantage and inequality. In a broad sense, the PIN programme was seen as a long-term community change strategy. |
| **YPP**  **Strange et al 2019** | Facilitators also empowered peer learning by encouraging parents to share their experiences of parenting and to learn from each other […]: ‘*One of the other mums… maybe even overheard the conversation… you'd have one mum come over and… maybe do this… I did this and it worked… and then you'd have another and another one and then all of a sudden you have this group of people trying to help you. And it's nice.*’ […] Facilitator engagement initiated learning experiences for the parents they were talking to and for other parents listening and observing. ‘*I really like seeing other people’s parenting... you learn what you want to do and what you don’t want to do… like when you see it in front of you… and I’ve learned a lot… just not from me asking for myself… but overhearing her (facilitator) from other parents asking her.’* |
| Strange et al., 2019 | Parents developed skills and knowledge through a variety of ways. Facilitators role-modelled positive parenting behaviors and interactions within the group, and used questions as opportunities for group discussions. These informal strategies and activities provided incidental learning opportunities that were effective, and have been reported elsewhere as more valuable in developing parenting skills compared to formal sessions. |
| Strange et al., 2019 | The parents co-design with staff the topics for talks (such as age related sleeping, feeding, ..), incursions (such as health and education visitors) and excursions (community activities such as library, parks, swimming and water safety lessons). ‘… After so many weeks we’ll do a Post-It note… what they’ve enjoyed about the group… is there anything to change? Part of our planning for the term is based on what ideas of parents come up with those things.’ For example, one parent was particularly concerned about car seats for infants as she felt some parents were not using correct car seating but did not want to raise this issue within the group. The confidential note system enabled her to raise the issue.” |
| **Baby and Us**  Webpage NHS Scotland Early Intervention Framework | Whilst there is a structure to the content of the programme, it is modifiable to an extent, parents can bring up topics of conversation and give one another support and ideas. Liz feels it differs from other programmes as it allows parents to reflect on their own experience of being parented, think about it from their perspective of being a parent and then also think about it from the child's perspective. |

1. **two CMOC’s belonging to the Programme Theory on ‘breaking the cycle’**

| **Mellow Babies**  Davidson et al., 2023 | Parents consistently indicated that the “Life Stories” session, a session strategically placed mid-way through the program, was a powerful intervention component that may drive change. The opportunity to formulate and reclaim the narrative of one’s own life experiences after establishing a trusting relationship within the group provided parents with a safe space to process and re-conceptualize their difficulties. For some parents, this also functioned as a turning point, leading to an emotional shift from which they felt they could “move forward” (Johnstone, 2018; Redhead et al., 2015). Specifically, parents indicated that the reflective elements of MB allowed them to become aware of past mistakes and maladaptive behaviors both of themselves, their parents, and their (past) romantic partners. This finding aligns with existing evidence suggesting that the provision of reflective opportunities may actively contribute to the prevention of the intergenerational transmission of psychosocial adversity (Schoon & Melis, 2019) |
| --- | --- |
| Davidson et al., 2023 | The nature of these discussions enabled parents to increase their understanding of complex interpersonal relationships and, in some cases, to recognize that they had been a victim (n = 6) or perpetrator (n = 2) of abuse in romantic relationships, “I was so blinded by my ex I didn’t even realize what he was doing was wrong” [Mum 22]. Both victim and perpetrator reflections indicated that understanding the patterns of abusive behavior made them feel more confident that in the future they would be able to avoid similar relationships, “*if the same thing was to happen again, I would know the cues and I would know how to spot it” [Mum 37]; “it’s something I’ll be able to combat now*” [Dad 1]. |
| Scourfield et al., 2016 | This emphasis on processing of difficult past experiences has several purposes. Firstly, it is designed to influence the capacity for attachment: ‘nurturing the parent to enable the parent to nurture the child’, […]. Secondly, and more pragmatically, it maintains investment in the programme. Allowing parents to talk about their own lives and difficulties they have experienced originally came into Mellow Parenting following feedback from mothers who had originally attended a programme with a more exclusive focus on behaviour management. These mothers wanted space to deal with their own difficulties in addition to learning parenting skills. […] The third purpose is that a ‘closed and contained’ group allows [parents] to build strong relationships with each other. Fourthly, these reflective discussions allow for difficult but important issues such as mental health and domestic violence to be aired, discussed and made sense of. |
| McGilloway, 2012 | it appears that under conditions of stress (including busy lives, bereavement, illness, separation from partner), parents reverted to using previous parenting behaviours and abandoned their newly learned skills, which still required a considerable level of conscious effort for these parents. This difficulty in perseverance suggests that these parents had not yet consolidated the skills into their lives in a habitual manner. However, once the stressful period had subsided, or parents had learned to cope with it, they were then able to re-introduce the parenting skills. This pattern suggests that the application of new parenting skills places extra demands on some parents, as it does not easily become second-nature to them but reassuringly, their subsequent recovery demonstrates that they had not lost the skills or their newly acquired knowledge |
| Levi, 2019 | […] improvements in maternal mental health and parenting confidence are consistent with approaches that suggest that parenting interventions should focus on both parent and child wellbeing (Alvarez et al., 2015), particularly when families are targeted on the basis of parental risk factors. Moreover, high parenting confidence may act as a buffer against factors such as parental depression, stress, relationship difficulties, and compromised child development, though the direction of influence is unclear. |
| Penehira & Doherty, 2012 | *Possibly the element where the participants had the opportunity to really look at their past and their present and have an opportunity to connect to their present practice of parenting and understand possibly why they do the things they do. I think that unless you have an understanding of how you got to where you are, it is quite difficult to move past that.* |
| Scourfield et al., 2016 | The Going Mellow manual cites the ‘Adult Attachment Interview’ (van Ijzendoorn, 1995) to support the idea that successful attachment to a child is more likely if a parent has an autonomous state of mind (which is seen as analogous to a secure attachment in childhood). It is important to know that it is not just whether you had a good childhood that defines your state of mind with respect to attachment, but whether you have been able to make sense of this. Parents who have had a very rough time as children can still develop what is known as “earned” security. |
|  | […] it is recognised that improved attachment is the aim. It is suggested, however, that this might perhaps be achieved in a slightly different way from how it is envisaged in formal programme theory [ i.e. repairing damaged attachment style of the parent]. It is possible that a warm, respectful, nurturing atmosphere is created and maintained by the morning session, which then opens fathers to learning more practical lessons. |
| Scourfield, 2014 | Those families most in need of support for managing their child’s behaviour are often not ready to consider and implement the suggested strategies because of their own issues, and so continue to fail to follow behavioural programmes. Mellow Parenting aims to reach these parents by providing a more nurturing context in which to develop their own relationships and their own skills alongside applying those to the relationship with their child. |
| **PIN**  McGilloway, 2012 | it appears that under conditions of stress (including busy lives, bereavement, illness, separation from partner), parents reverted to using previous parenting behaviours and abandoned their newly learned skills, which still required a considerable level of conscious effort for these parents. This difficulty in perseverance suggests that these parents had not yet consolidated the skills into their lives in a habitual manner. However, once the stressful period had subsided, or parents had learned to cope with it, they were then able to re-introduce the parenting skills. This pattern suggests that the application of new parenting skills places extra demands on some parents, as it does not easily become second-nature to them but reassuringly, their subsequent recovery demonstrates that they had not lost the skills or their newly acquired knowledge |
| Hickey 2024 | the findings reported here, indicate that a lack of perceived need, particularly during the toddler years, was a barrier to parent retention in the PIN programme. These considerations may have been intensified by the requirement for parents to attend a relatively lengthy (12-week) programme, as well as the presence of competing demands (e.g. return to work). |
| **Mother Baby Nurture**  Cooke et al., 2023 | A recent meta-analysis [of behavioral parenting programs found] little to no post-intervention reduction in parenting stress (and depression) which are known risk-factors related to child maltreatment. Chen & Chan (2016) concluded that programs commonly aim to increase parenting skills, however, these behaviours deteriorate when under stress. […] Parenting stress disrupts the caregivers capacity to attend to the infant (Doiron & Stack, 2017) and is a known risk factor for child maltreatment. |
| Cooke et al., 2023 | As the mother seeks to care for her infant, the facilitators may notice that the mother expresses strong emotions, ambivalent or negative feelings in what she says about her infant or in the way she responds or handles him. The facilitators emotionally contain the expressed state, allowing the mother to talk about her experiences, without fear of abandonment, intrusion, or criticism. A mother who is able to articulate her longing, or to mourn her loss or express her anger or despair within the context of a nurturing relationship may become clearer about her relational history and more emotionally available and sensitive to her infant [30, 64, 65]. In the safety of the supportive relationship, the mother may become more able to mentalize emotionally charged events and this lowers her epistemic vigilance [24]. This capacity will support her to revisit the difficult experience in a more resourceful way, giving her opportunity to better understand and integrate the feelings that threaten the developing attachment relationship [24, 30]. Well-regulated affect between the dyad can be internalised into the child’s developing internal working model and ‘secure base’ attachment relationship [66], reducing the risk of intergenerational transmission [57, 67]. The mother learns how to provide contingent responses to the infant, so the infant can register his mental states as a coherent part of himself, rather than as random or alien [68]. This learning is possibly through the development of epistemic trust [24] and is then applied in everyday life. |
| Cooke et al., 2023 | The heightened distress experienced by some mothers is likely to be related to experiences of early attachment relationships and insecurities activate by the presence of the infant, leading to a high level of unexplained arousal, accompanying strong feelings of fear of abandonment or a sense of overwhelm (Nijssens et al., 2018). Once trusted relationships are established in the MBN group, a core component of the program is to mentalize these real-time affective experiences, distorted thinking, and live encounters with their infant in an effort to support the mother to provide healthier relational dynamics |
| **Baby and Us**  Webpage NHS Scotland | [trainer] feels [Baby and Us] differs from other programmes as it allows parents to reflect on their own experience of being parented, think about it from their perspective of being a parent and then also think about it from the child's perspective. |

**CMOC 12 Feeling better**

As parents with mental health challenges (C) become more free from their past, and the trust and mutual support within the group grows (M resource), they feel validated and back in control of their life and of their parenting choices (Mresp), leading to improvements in self-esteem *(personal empowerment)* and mental health (e.g., reduced feelings of stress, depression and anxiety, more calmness to cope with parenting challenges and more adaptive coping skills) (O).

| **Mellow Babies**  Davidson et al., 2023 | perceived improvements in mental health led to a reduction in parenting-induced stress and enabled them to access more adaptive coping skills and emotion regulation strategies (n = 33), “*how I’m handling myself whenever situations come up, even [with] the two older ones, I’ve been more relaxed in my approach with them and their behavior*” [Dad 1]. |
| --- | --- |
| Davidson et al., 2023 | Both mothers and fathers experienced an improvement in their mental health following attendance at MB (n = 43), including positive changes in their mood, anxiety and self-esteem; one to such an extent that they were advised to discontinue their anti-depressant medication. Maternal reflections (n = 15) highlighted the role of MB in enabling them to reconnect with neglected aspects of their identity beyond motherhood and mental health challenges, “*I’ve gained a bit of me back. I thought - “That’s it, I don’t matter anymore”. But you can only last so long like that and you can feel lonely, sad, depressed. I feel a hell of a lot better now*”. |
| Davidson et al., 2023 | As a consequence, parents reported feeling more confident both in their daily life activities, “*I didn’t like going to big public spaces, I just felt everybody was talking about me and looking at me”* [Dad 16] and in their parenting skills following MB attendance, “*I used to ask my mum for a lot of help - I felt I wasn’t able to do it myself whereas now I feel I can”* [Mum 46]. This confidence boost contributed to reduced isolation via increased social connectedness and ability to engage with the community for both mothers and fathers (n = 58), “*I’m more sociable, I have more friends, I can talk to people. I’m back out in the community again rather than being so isolated and having nobody* |
| Davidson et al., 2023 | Parents indicated that the value of MB did not lie in “prescribing” change for them but in making them realize that circumstances can change and empowering them to generate positive changes for themselves. |
| Scourfield, 2014 | [the group] *helped me quite a lot when it was there, because it brought me out of my depression quite quick, and I’ve got two or three good mates that I keep in touch with, sometimes. But if I’ve got a problem, I’ll give Neil a phone and have a blether. (Brian, father)* |
| Doherty, 2012 | *Before I used to beat myself up – oh I’m struggling, got no job, got no future, that was the outlook before… it has helped me refocus… I’m helping my kids on a daily basis, I get them off to school, I feed them, I clean them, I love them, you know, only through the conversations that we have here and sharing our life stories and experiences was I able to reconnect with those things and try and formulate a plan to move forward…I had to come here for people to say “look bro, you are doing all these things and you don’t even realise”, and so that was a huge growth thing for me, giving myself a pat on the back, recognizing I’m putting in some good work,…and take this into the future with my kids* |
| Penehira & Doherty, 2012 | *Empowering for all of us as parents. Like you do have a choice. Because with me it was that I felt like I didn’t have a choice because I was a solo mother with my two boys and just feeling sorry for myself and letting them get away with a lot of stuff.… It shows you don’t have to be a victim to your children … now I have actually learnt how to say “no” and mean it.* |
| Raouna, 2021 | At-risk’ parents and, specifically, parents experiencing deterioration of their mental health during the first postnatal months, can find it difficult to focus on their infant, notice their signals and interests, and respond appropriately [65, 66]. Providing support to parents to reduce the levels of their symptoms during the MB intervention can, therefore, also allow them to tune-in better to their babies’ emotional and practical needs and positively impact the way they perceive the quality of their relationship |
| **Parent Infant Program** Hickey, 2023 | Positive effects on the health and well-being of parents were also reported, including significant post-intervention improvements in levels of depression. “*I feel like I am calmer now […] I used to not have five minutes for myself because everything was go, go, go.”* |
| ENRICH | Integrated and multidisciplinary supports which are delivered in community-based settings can result in sustained improvements in parenting self-efficacy. Parents who have a greater sense of mastery and confidence tend to experience more positive mental health and use more positive parenting strategies. Greater parenting self-efficacy has also been associated with positive socioemotional wellbeing in children. Universal, group-based early parenting programme may have beneficial outcomes in terms of child temperament and problem-solving skills. However, these benefits accrued to higher functioning families and no intervention effects were observed for high-risk parents and their young children. More research is needed to explore the kinds of adaptations and supports which are required to ensure that all families and children are supported to achieve their full potential. |
| **Mother Baby Nurture**  Cooke et al., 2023 | It is possible that strengthening and supporting the infant–mother relationship and reflective functioning in the MBN group may have lessened the mothers’ mental health difficulties. [..] parent education programs have shown limited evidence of reducing parental depression and stress (Chen & Chan, 2016). An increase in the mother’s sense of confidence was also noted in the qualitative responses. The authors propose that the relational experience of the group has translated to greater parenting confidence via increased epistemic trust. |
| Cooke et al., 2023 | The group process acts as a holding environment for the vulnerable mother […] When a member of the group shares an affective state, the containing experience of marked mirroring can be amplified and nuanced as the multiple members provide a “hall of mirrors” response that offers differing affective intensity and hues [47]. The group can also offer some distance when a member listens to another’s experience, she can gain insight into aspects of her own internal world that may have been previously obscured. The process of identifying one’s own experience within the story of another member is both validating as well as normalising, alleviating feelings of isolation and shame. Establishing the service within the community instead of hospital setting, also helps destigmatise their experience. |

1. **Three CMOC’s belonging to the Programme Theory on ‘Connection with baby’**

**CMOC 13 Baby’s development drives change**

As parents of children who have not experienced much social interaction, feel supported and nurtured (C), the stimulating environment, social opportunities for parent and baby, and the positive labeling of baby’s natural development within the group (Mresource) encourage parents’ positive attitude towards baby (O), and confidence in their parenting abilities (O), further reducing parenting stress (O).

| **Mellow Babies**  Davidson et al., 2023 | Several parents witnessed a positive widening of their infant’s social world post-intervention, with some parents (n = 10) reporting that their baby had never played with other children before attending MB. Parents, in the vast majority mothers, highlighted that the attendance of their children at the childcare group that runs parallel to the MB group had benefitted their infant’s social development ( n = 35), mental health (n = 25) and confidence (n = 9), “*my baby is very sociable now whereas beforehand he would have been strange with people*” [Mum 25]; “*he learned how to smile*” [Mum 34]. |
| --- | --- |
| Raouna, 2021 | this study only assessed the intervention effects immediately after MB completion, making it impossible to reach conclusions for the long-term effects of MB and possibly underestimating its effects in areas that need more time to emerge, such as child developmental outcomes. |
| Peniha & Doherty, 2012 | Facilitators commented that improvement in children’s behaviours was actually a very powerful encouragement for parents’ change: “*Of the children, huge, really huge. And then it was really interesting to see how the parents interpreted that. Because the children’s behaviour had improved, their level of hassle, their level of stress around parenting reduced quite significantly. [..] We will be getting the teachers and caregivers who are going to be looking after them to do some of the behaviour and development questionnaires, pre and post as well. Because it seems to be a key and if others can be instrumental in supporting change with the children while others are working with the mothers together, it could be even more powerful than it has been*.” |
|  | […] it is recognised that improved attachment is the aim. It is suggested, however, that this might perhaps be achieved in a slightly different way from how it is envisaged in formal programme theory [ i.e. repairing damaged attachment style of the parent]. It is possible that a warm, respectful, nurturing atmosphere is created and maintained by the morning session, which then opens up fathers to learning more practical lessons. |
| **Parent Infant Program**  Leckey et al., 2019 | Having the babies present during the programme was viewed as hugely beneficial both in terms of seeing how other infants were in the group were developing at different rates and also for allowing mothers to put apply new skills with their own babies: “*Week to week when you’re physically seeing the babies there, so I got to see other people’s babies and how they were growing and developing and they fed back what they could see with [my baby]. Like that she was changing, growing, getting bigger, had learned how to do something* ::: |
| **Young Parent Program**  Strange et al., 2019 | Parents wanted the socialisation for their child with other children, and enjoyed learning about stimulation and play, activities they could replicate at home. These activities supported the importance of a home learning environment for children. “*I think everything they do here is good. They have this sensory day… so the kids can touch things and work out with their fingers and what things feel like…* |
| Strange et al., 2019 | Facilitators provided age appropriate activities for the children. This included baby massage and sensory mats for the infants, toddler outdoor and play-based learning activities, and sing-a-longs. Although some parents were reluctant to sing along with the facilitators, in time, more of the parents joined in: ‘*All these nursery rhymes I’d never heard before… so I had to learn them…most of us sing them now.* |
| **MOther Baby Nurture**  Cooke et al., 2023 | The infants developing sense of subjective self, as a separate entity from the caregiver, is a central organising process of psychological development [63, 69]. The group provides a transitional space for the infant [70], where he can observe those around him, noticing similarities and differences, and feel safe enough to explore new experiences of self. This can be a powerful learning opportunity especially for a socially isolated mother and baby, as it offers a space in which alternate expressions of thinking and feeling can be experienced and offered. |
| Cooke et al., 2023 | Ways of being together become imbedded in the infant’s procedural memory, forming an internal representation of how others relate to him and how he relates to others. These repeated “serve and return” experiences between the infant and others, most especially their caregivers, form the infant’s internal working model. |
| Cooke et al., 2023 | Engaging the mother in isolation misses an opportunity to directly contribute to the infant’s development, the quality of the mother-infant interactions and the promotion of infant mental health [34]. Paradoxically due to neuroplasticity, the vulnerable infant is the most receptive and adaptive member in the dyad, making them a potent agent of change in the relationship. Stirred by the enlivened infant, the mother’s attachment systems can be activated, creating opportunity for reorganisation of internal representations, role and emerging attachment patterns [26], as well as inhibiting disorganised attachment in infants |
| Cooke et al., 2023 | Being a new experience, the mother’s thoughts and feelings towards her newborn baby and her new caring role are less established and likely to be more flexible. The mother’s patterns of behaviour are still in formation and responses are not yet predictable and anticipated, allowing opportunity for flexibility and change. Consequently, the MBN program is offered within the first six months post birth to seize this opportunity of flexibility in the mother-infant dyad. |

**CMOC 14 Learning about parenting**

When disadvantaged parents who have little experience of engaging in educational, playful activities with their children, and little knowledge of positive parenting skills, reading baby’s cues or developmental milestones (C), attend group-based programmes that provide hands-on opportunities for parents to learn, exchanging of advice with other parents, and positive role modelling (Mresource), parents are more likely to change their parenting practices and take a more proactive role in their child’s development (O) due to increased confidence and feelings of empowerment related to their parenting skills (Mresp).

|  |  |
| --- | --- |
| **Parent Infant Program**  Hickey et al., 2023 | Observations of parent–child interactions in the home demonstrated that, post-intervention, parents were using significantly more positive parenting skills, whilst use of negative and critical parenting was significantly reduced. |
| Leckey et al., 2019 | Some mothers indicated that the program provided them with useful information on common parenting issues and increased their confidence in their abilities to predict their infant’s needs and encourage their development. Notably, first-time mothers, with little practical experience in caring for babies, felt that they benefitted hugely from the program: *“I didn’t know where to start with [baby] and the program helped me a lot* […]”second-time mothers also reported gaining new knowledge and skills around infant health and development. For example, one parent reported how the programme provided her with additional information on how to respond promptly and appropriately to her infant’s cues: ::: *I am aware more about stuff. Because before it was like why are they crying? I will just try everything. And then I got the facial cues and the tongue and stuff like that so I was able to do things like feed her before she’d start crying* |
| Leckey et al., 2019 | the challenges associated with modifying parenting behaviour and attitudes was widely acknowledged by facilitators, both in instances of ‘hard to reach’ mothers, but also for ‘middle class’ mothers with preconceived, or idealised parenting beliefs: “*Definitely where a person is coming from, what their experience as a child has been effects how they parent ::: . our hard to reach mums would be particularly like that ::: They are the hardest to change :::”* |
| ENRICH | Parents who have a greater sense of efficacy in parenting tend to experience more positive mental health and use more sensitive and responsive parenting strategies. Greater parenting self-efficacy has also been associated with positive socioemotional wellbeing in children. |
| Leckey et al., 2019 | In particular, the inclusion of wraparound workshops such as baby massage, weaning and play and development, gave mothers the impetus to anticipate their infant’s needs and to encourage their development: “We had a lot of direction around upcoming milestones and what you would need to be focusing on to make sure that your child is going to achieve them ::: about brain development and I got a huge amount out of it and so did [baby]. |
| Puckering 2008/2009 | All the activities are free or use inexpensive materials so that the family can continue to use them outside the group. Parents, who have never experienced play in their own lives and never played with their child, can find out how much fun messy play or simple craft activities can be. Outings to the local children’s library or the park also open the doors to all the local facilities, of which the parents may have been unaware or too lacking in confidence to try out. |
| Leckey et al., 2019 | Following programme participation, mothers spoke of being more sensitive and responsive to their infants’ emotional needs and reported having a more proactive role in their development. […]. Equally, facilitators remarked on increased parental confidence and self-esteem as well as greater use of positive communication between PIN, thereby suggesting the programme empowered mothers in their role as parents. The facilitators also observed greater mother–child interaction at clinic visits. |
| Leckey et al., 2019 | The groups provided a source of informal knowledge and support where mothers could openly express their frustration or anxiety and exchange advice on parenting needs. […]. These new connections and interactions helped to promote confidence in their parenting techniques: “Meeting new mums and getting information that you don’t know as a first time mother ::: because you think you’re the only one so it’s good to be able to be able to talk to people |
| McGilloway, 2012 | Parents also attributed their success in changing their parenting strategies to feelings of increased confidence in their competency as a parent. |
| **Young Parent Program**  Strange et al., 2019 | Facilitators felt that parent confidence had increased over the course of the program and this was regarded as an important outcome. Parents did not spontaneously use the term ‘confidence’ in the interviews. However, when asked if attending the program had influenced their parenting confidence they all shared that they were more confident. One parent spoke of being more comfortable as a parent: ‘*It’s made me a lot more comfortable… as a parent and a friend… like I was saying I was nervous coming here… but now… I feel like I can go out and do more things without having that worry of being nervous I guess.’* (P6 child 6 months) |
| Strange et al., 2019 | In addition to traumatic backgrounds experienced by several parents, most parents had little to no experience with infants prior to becoming a parent and many had limited support networks with parenting experience. Observing facilitators role modelling responses to infants as well as peer learning helped parents to ‘tune in’ and develop ways of positively interacting and stimulating their child (32). One parent described how she learned about ‘tummy time’ and this helped her child to learn to roll over: ‘*Before she was five-and-a-half months old… she never rolled… she never did tummy time… she never did any of that because she just didn’t want to and I just didn’t push her. She started coming here and probably the second time we’re here she rolled. Her skills are developing*.’ |
| Strange et al., 2019 | Parents developed skills and knowledge through a variety of ways. Facilitators role-modelled positive parenting behaviours and interactions within the group, and used questions as opportunities for group discussions. These informal strategies and activities provided incidental learning opportunities that were effective, and have been reported elsewhere as more valuable in developing parenting skills compared to formal sessions (30). A strengths-based approach that focused on what parents were doing well, and the opportunity to talk with others helped parents to feel reassured and more confident in their parenting, and is consistent with other authors (3, 4, 12, 31). One facilitator described ‘*picking up positives that someone is doing’*. (F1) |
| **Baby and Us**  Harwood et al., 2022 | Parents reported improvements in their goals, in mental wellbeing, self-acceptance, learning, knowledge and parenting confidence, all with a large effect sizes [..] parents rated that they increased their understanding and skills in positive parenting, increased parental confidence and felt more equipped to use their learning in practice. |

**CMOC 15 Connection**

When parents who feel unsure about their connection with their baby and whether their baby loves them (C), receive positive feedback on their parenting style through looking at video-taped interaction or by receiving group facilitators’ or other parents’ feedback (M resource), they will gain greater empathy and understanding of the child’s needs and responses to their interactions (M response). Love and a feeling of connection are fostered (M resp), leading to improved parent-baby interaction (O).

| **Mellow babies**  Sourfield et al., 2016 | Fathers who had taken part in Mellow Dads claimed the programme had made a positive difference to the ways in which they interacted with their children […] one father said he felt that he was more involved in the play with his child these days, and had many more ideas for activities he could do with his child. He had taken some books and toys along to his contact session for his baby to play with, and we were told that previously he had usually or even always turned up to contact empty-handed |
| --- | --- |
| Puckering 2010 | Video assessments, using the Mellow Parenting Coding system, found that observed positive mother-infant interactions increased significantly following the intervention group as compared with the control group. Conversely, relative to the control group, the lower levels of total observed negative interaction approached a significant level in the intervention group. |
| Davidson et al., 2023 | *“I wasn’t sure about being around him, I didn’t feel I had a connection with him, I didn’t feel there was a bond there with him, I wanted to foster him out at the beginning – but now, I just love him to bits*”. |
| Raouna | […] parents […] reported increased perceived connectedness with their child, moving closer towards their ‘ideal’ relationship over the course of MB. […] Also, over half of the parents involved with child protection services witnessed a de-escalation of their case by the end of the group. |
| Penehira & Doherty, 2012 | Learning specific behaviour management skills such as: listening to children; providing children with options; and managing a number of siblings in one family, provided participants with the necessary tools to make very concrete changes to their parenting. “*It is really life changing because it is life changing for my kids as well because they see the results in me because Mum is not yelling all the time. Mum is not frustrated all the time. [..] when I first came here [baby name] just really didn’t like being around me. […] So they taught me how to balance things that it is not all about your job and it is not all about what you can give your kids but the most important thing is to spend time with them. My son is not an angel but now he wants me, which is a great feeling*.’ |
| Puckering, 2010 | [..] the video workshop had enabled mothers to develop empathy with their child in wondering, ‘*how it feels for him/her?’ “Exercises like that made me put myself into the bairn’s [child’s] shoes and I understood what I would be able to do and wouldn’t be able to make her more comfortable or uncomfortable*” |
| **Parent Infant Program**  Leckey et al., 2019 | Following programme participation, mothers spoke of being more sensitive and responsive to their infants’ emotional needs and reported having a more proactive role in their development. Techniques such as singing, playing and reading to their infant were introduced earlier in order to encourage and stimulate their infant’s socioemotional and cognitive development. […]. The facilitators also observed greater mother–child interaction at clinic visits. |
| Leckey et al., 2019 | Initially, many mothers believed that their baby could not understand, or benefit, from interaction at such a young age. As a consequence of programme participation, many felt encouraged to communicate more with their baby in order to promote their language and cognitive skills: “*Like your connection with your child. I don’t think people realise, at such an early stage, how important say the parentese and stuff like that, even talking to your baby, how important it is for their development. (P22) [..] How you interact and communicate with your child has a big impact on how development is ::: He is very curious, he’s very interested in things and I’m sure that has been because we’ve been more focused on interacting with him a lot more.”* |
| **Young Parent Program**  Strange et al., 2019 | Parents learned through the group how to ‘tune in’ to their child’s needs. An attuned parent is responsive to their infant, soothing when needed and mirroring pleasure and affect so the child feels secure in the care (32). One facilitator illustrated the infant ‘tuning in’ and encouragement and support needed for parents who had experienced trauma and rejection in their own lives. ‘*We get a lot of these first-time mums… because they have had quite a lot of trauma in their own background and rejection… as we know… comes out in their parenting. So sometimes a comment can be made (by parent)… “Oh, she just doesn’t like me. She never smiles at me.” and just by having someone there to say… “Well… look at her… she's actually looking at you. She needs you. You're a great mum.” So… all of that promotes attachment and caring and bonding and picking up then on the needs of the child which strengthens the (parent and child) relationship* |
| **Mother Baby Nurture**  Cooke et al., 2023 | “*This group totally changed motherhood for me. I stopped worrying so much about what ‘should’ be happening and connected with my baby, learnt to trust myself and enjoyed the ‘dance’ instead. I couldn’t be more grateful because I know it has changed [baby name] life now and in the future. Great program. Learnt so much and felt my confidence grow. Felt well supported at all times. Very true to its name—I felt nurtured, listened to, and supported.* |
| Cooke et al., 2023 | [..] responding to the infant and holding him in mind as a thinking, feeling being is a central aspect of MBN. Such interventions support the mother’s capacity for perspective-taking, beyond her own experience and adult concerns, to consider the perspective and experience of her infant [37]. |
| Cooke et al., 2023 | Through repetition, modelling curiosity about internal states is transmitted to the mothers, encouraging them to consider their own and their infants’ internal states. This curiosity and openness in thinking helps to develop the skill of metacognition, so rather than being ‘in it’ the mother is able to think ‘on it’ which enables her to examine her internal working model and how she views the intentions of her child and her own self [60]. Through practicing this type of perspective taking, the mother’s mentalizing capacity is stretched and strengthened. This way of being, once nurtured in the group can continue to develop beyond the life of the group and is passed forward through the infant-parent relationship. |

1. **One CMOC belonging to the Programme Theory on ‘Spreading their wings’**

**CMOC 16 Spreading their wings**

If parents acquire/develop sufficient trust in their personal network and self-confidence through the completion of a parenting program (C), then they have hope for an improved future (M resp). The support and guidance from the group facilitators and other parents (Mres) empowers them to seek paid employment or formal education (O). Additionally, they also have increased confidence to continue expanding their social connectedness, seek help when going through difficult periods in their lives, and feel calmer (M resp) when engaging with external organisations such as their employers, schools, social services or new parenting groups (O)

**CMOC 16a Not ready yet**

If parents do not acquire sufficient self-confidence in their parenting abilities by the end of the program or have insecurities associated with their future (C), they may experience fear and a sense of loss of the safe group environment (Resp); longer periods of support in some way or another may be needed (Mresource), in order to negate feelings of shame and insecurity and to help parents navigate future parenting challenges and crises (O).

| **Mellow Babies**  Davidson et al., 2023 | When considering their plans and goals following MB, several parents expressed a desire for the group to be extended (n = 15) or to repeat the group entirely (n = 13), *“I would give anything to come back to another Mellow Dads group” [Dad 12*]. There were both maternal and paternal reports wishing the group would “go on forever”, with some parents indicating that their desire to repeat the group was embedded in the opportunity to learn more (n = 3), “extra learning, that’s what I’ll miss the most” [Dad 11], or to make sure they had not missed any important elements (n = 4), “when they start the Mellow Dads up again I’ll probably ask to attend again, just in case there’s anything I’ve maybe missed” [Dad 12]. A few parents expressed apprehension and a lack of confidence regarding their future (n = 10), expressing doubts about the lasting effects of MB on their lives and feeling insecure without the safe haven that the group offered to them, “I’ve got a bit more confidence but it’s not enough to keep me going, as soon as this group’s finished I’ll probably be going back in again. I don’t know what I’ll do without this group, I don’t want it to finish”  On the other hand, some parents shared that the group provided them with motivation and hope for their futures, “I don’t want to be on the dole all my life and this group has said “you can get a job and make your daughter proud”, no-one’s ever said that. No-one’s ever pushed me towards doing it” [Mum 13]. Approximately half of the parents expressed a desire to continue developing autonomy through the acquisition of new skills either by moving into education (n = 10) or seeking employment (n = 22). Parents reported feeling empowered by the support and guidance received from other group members and specifically group facilitators to explore employment and education opportunities available to them post-intervention, “*it’s really, really helped me, I mean if you’d said to me a year ago “In a year’s time you’re going to be making an appointment with the Job Centre, I’d have went “No, I’m not!”*’ [Dad 16]; “*I’ve been talking to [the facilitator] and they’re going to put me forward to do a child care course”* [Mum 42]; *“it’s sort of showed me what I want to do. I’m going to try and get into uni*” [Dad 18]. *“Probably at the start of the group I had never, ever seen a future for me, I never – I found it hard to look into the future, I didn’t – it’s hard to explain but I just didn’t see anything, I was so just to just feeling numb but now I’m much more confident and I see a really good future for me and the girls, so it’s just me and my girls. Maybe getting a job in like six months’ time, providing for them, just being safe and happy” [Mum* 21]  Acknowledging their need for continued personal and parenting support, some parents also planned to move onto Mellow Toddlers (n = 5) within the same community setting, while many parents (n = 20) appeared to have the confidence to re-engage with the wider community by attending other support and parenting groups, “*I am planning on taking the kids to other toddler groups in the area”* [Mum 26]. Lastly, the majority of mothers and fathers (n = 41) expressed a desire to maintain the friendships acquired throughout the group; indicating a sustained increase in proactive social connectedness, “*I’m going to keep in touch with most of them here. I hope to try to organize an actual dads’ group myself - go to the soft play or go swimming*” [Dad 11].  The positive impact of MB in interpersonal relationships extended upon the parent-child relationship and permeated the wider family context. Parents reflected on improved relationships within the family home as a result of attending MB, including with their own parents and other children. There were also parental reports of adopting a calmer approach when engaging with schools and social services. In some cases, the impact of increased parental confidence also expanded in other aspects of their lives, exemplified by an increased desire to engage with further education, employment, or sustain acquired social connections. In this way, MB appeared to have an attachment function, providing parents with a secure base from which they felt capable of further exploring their world and the opportunities available.  As a consequence, parents reported feeling more confident both in their daily life activities, “I didn’t like going to big public spaces, I just felt everybody was talking about me and looking at me” [Dad 16] and in their parenting skills following MB attendance, “I used to ask my mum for a lot of help - I felt I wasn’t able to do it myself whereas now I feel I can” [Mum 46]. This confidence boost contributed to reduced isolation via increased social connectedness and ability to engage with the community for both mothers and fathers (n = 58), “I’m more sociable, I have more friends, I can talk to people. I’m back out in the community again rather than being so isolated and having nobody  Specifically for parents involved with social services, MB was perceived as a space that “allowed” them to parent their child, “I didn’t feel like I was being watched or judged. I could just be mum to her” [..]. For some parents […], this positive relationship established with the facilitators created a vehicle to re-engage with services from which they had previously felt marginalized, creating a positive snowball effect of improved relationships with healthcare related professionals. |
| --- | --- |
| Scourfield et al., 2016 | This was especially so for those fathers who chose to attend the monthly post-Mellow Dads support group. Some of these men had in fact requested the group be set up, to offer them on-going support, because they were missing the group. The excerpts below illustrate the importance of ongoing support from other fathers. The ‘Neil’ mentioned by Brian is another father from the group he attended. *It helped me quite a lot when it was there, because it brought me out of my depression quite quick, and I've got two or three good mates that I keep in touch with, sometimes. But if I've got a problem, I'll give Neil a phone and have a blether.1 [(Brian, father)]. You know, we were in limbo after it finished, because we enjoyed going there that much. So we decided to start this group, and carry on. [(Eddie, father)]* |
| Levi, 2019 | 64.5% (n = 78) strongly agreed with the statement “*I feel confident in asking for help should I need it*”. Data from facilitators on uptake of further service was available for n = 61 participants (see Supplementary Table 3). Facilitators indicated that 34% (n = 55) of mothers who engaged until the end of the intervention expressed an intention to engage in further group-based community services and 3% were referred to specialised one-to-one family support (n = 5). |
| Raouna, 2021 | The feedback questionnaire indicates that the vast majority of participants intended to remain engaged with parenting services and reported feeling more confident to seek professional help should they need it. This suggests that MB participants developed trusting relationships with group practitioners and services, reducing access barriers usually associated with help-seeking during the perinatal period [49, 50]. We note that MB promotes the continued involvement of parents with services, and group facilitators provide tailored support to parents upon program completion, directing them to relevant self-development and family-focused initiatives if deemed appropriate. |
| Puckering 2008/2009 | Strengths-based support from the group helped [a mother] to recognise how her behaviour was going to result in her losing a third child. […] She began to talk about her baby and […] began to caress the bump during the groups. She was set up with an advocate and mentor and developed extremely good relationships with them. Her mentor was honest with her, but kind and attended appointments with her to visit housing and other services. |
| **Parent and infant program**  McGilloway, 2012 | Introducing post-course support for parents at stressful junctures was also suggested. Although some parents sought support from the group facilitators during relapse, others reported that they would like the group facilitators to take a more proactive role in contacting them, or in establishing intermittent follow-up meetings in order to offset any emergent feelings of inadequacy and shame. |
| Hickey et al., 2023 | *I think that you may not have as many mums falling through the net. [. . .] I think it is actually linking up a lot more people and even the community centres [and] I think there is a lot more communication in the services.* (I2, program facilitator) |
| Hickey et al., 2021 | *“I say I would like that to be within the health centres if at all possible because for the families there, oh I was up in the health centre, it is a lovely place, [....] That demystifies what this health centre is. And if it is a case that they are going for any other checks or appointments, they know where it is, they know the system. And that fear factor is taken from them.”* |
| Hickey et al., 2020 | The PIN intervention may also have had potentially beneficial effects on service utilisation patterns […] Thus, integrating early parenting supports within a coordinated and multidisciplinary system of services may be beneficial in terms of promoting more cost-efficient engagement with primary care health services and, at the same time, priming parents’ engagement with community-based parenting supports. |
| **Mother Baby Nurture**  Cooke et al., 2023 | This confidence also extended to benefiting other family members: “The tools MBN has given me to be a confident mum and also to open up conversations with my partner to increase his confidence as a dad and to discuss how we wish to parent together”. |
